# Coordination of meristem and boundary functions by transcription factors in the SHOOT MERISTEMLESS regulatory network

**DOI:** 10.1242/dev.157081

**Published:** 2018-04-30

**Authors:** Simon Scofield, Alexander Murison, Angharad Jones, John Fozard, Mitsuhiro Aida, Leah R. Band, Malcolm Bennett, James A. H. Murray

**Affiliations:** 1 School of Biosciences, Cardiff University, Museum Avenue, Cardiff CF10 3AX, UK; 2 Princess Margaret Cancer Centre, University Health Network, Toronto, Ontario M5G 2M9, Canada; 3 Department of Computational and Systems Biology, John Innes Centre, Norwich NR4 7UH, UK; 4 International Research Organization for Advanced Science and Technology (IROAST) Kumamoto University, 2-39-1 Kurokami, Chuo-ku, Kumamoto 860-8555, Japan; 5 Centre for Plant Integrative Biology, Division of Plant and Crop Sciences, School of Biosciences, University of Nottingham, Loughborough LE12 5RD, UK; 6 Centre for Mathematical Medicine and Biology, School of Mathematical Sciences, University of Nottingham, Nottingham NG7 2RD, UK

**Keywords:** SHOOT MERISTEMLESS, Gene regulatory network, *Arabidopsis*, Homeodomain, Meristem, KNOX, Transcriptional regulation

## Abstract

The *Arabidopsis* homeodomain transcription factor SHOOT MERISTEMLESS (STM) is crucial for shoot apical meristem (SAM) function, yet the components and structure of the STM gene regulatory network (GRN) are largely unknown. Here, we show that transcriptional regulators are overrepresented among STM-regulated genes and, using these as GRN components in Bayesian network analysis, we infer STM GRN associations and reveal regulatory relationships between STM and factors involved in multiple aspects of SAM function. These include hormone regulation, TCP-mediated control of cell differentiation, AIL/PLT-mediated regulation of pluripotency and phyllotaxis, and specification of meristem-organ boundary zones via CUC1. We demonstrate a direct positive transcriptional feedback loop between STM and CUC1, despite their distinct expression patterns in the meristem and organ boundary, respectively. Our further finding that STM activates expression of the CUC1-targeting microRNA *miR164c* combined with mathematical modelling provides a potential solution for this apparent contradiction, demonstrating that these proposed regulatory interactions coupled with STM mobility could be sufficient to provide a mechanism for CUC1 localisation at the meristem-organ boundary. Our findings highlight the central role for the STM GRN in coordinating SAM functions.

## Abstract (precis)

**Highlighted Article:** The *Arabidopsis* transcription factor SHOOT MERISTEMLESS coordinates multiple aspects of meristem function by regulating the expression of other transcription factors with key roles in pluripotency and pattern formation.

## INTRODUCTION

Gene regulatory networks (GRNs) are employed in multicellular organisms to control cell-, tissue- and organ-type specification through transcriptional programming. Homeodomain (HD) proteins represent one class of developmental transcription factor (TF) that is conserved in metazoans and plants, and have key functions in the establishment and delineation of tissues in early development. In plants, where aerial organs are formed continuously throughout the life-cycle, establishment and maintenance of the shoot apical meristem (SAM) is controlled by the KNOX HD protein SHOOT MERISTEMLESS (STM), while organ primordia are specified at the meristem periphery by the accumulation of the phytohormone auxin, activation of auxin responses and resultant expression of organ-specific transcriptional regulators (Long et al., 1996; Byrne et al., 2002; Benková et al., 2003; Hay and Tsiantis, 2010; Tsuda and Hake, 2015). Delineation of the meristem from organ primordia involves the formation of a boundary region specified by the CUP-SHAPED COTYLEDON (CUC) transcription factors (Aida et al., 1999).

*STM* is a class-I KNOX gene that encodes a mobile TALE homeodomain transcription factor previously shown to be essential for development and sustained function of the SAM. Loss-of-function *stm* mutants show defects in SAM development and function, ranging from defective SAM organisation in weaker alleles such as *stm-2*, to abolition of SAM formation in strong alleles such as *stm-1* (Long et al., 1996; Clark et al., 1996). *STM* is expressed throughout the SAM but is downregulated in incipient organ primordia, coincident with the accumulation of auxin and the expression of organ-specific genes such as the R2R3 MYB transcription factor *AS1* and the LOB-domain protein AS2 (Ori et al., 2000; Byrne et al., 2002), which are known repressors of KNOX gene expression. Other factors that repress KNOX gene expression in leaf primordia include the TEOSINTE BRANCHED1/CYCLOIDEA/PCF1 (TCP) family of bHLH-type transcriptional regulators, which also function to promote leaf differentiation (Li, 2015).

Exclusion of STM from leaf primordia is important for proper differentiation, as ectopic expression of *STM* or other class-1 KNOX genes, such as *KNAT1/BP* and *KNAT2*, causes drastic phenotypic effects ranging from inhibition of cellular differentiation in leaves to the formation of ectopic shoot meristems (Chuck et al., 1996; Brand et al., 2002; Lenhard et al., 2002; Scofield et al., 2013). *STM* has been shown to promote the biosynthesis of cytokinin (CK) through activation of *ISOPENTYLTRANSFERASE7* (*IPT7*) gene expression (Yanai et al., 2005; Jasinski et al., 2005). KNOX genes in various species have also been implicated in the repression of gibberellic acid (GA) and brassinosteroid (BR) biosynthesis, and in the inhibition of auxin responses (Sakamoto et al., 2001; Hay et al., 2002; Bolduc and Hake, 2009; Tsuda et al., 2014; Bolduc et al., 2012). Hence, KNOX genes such as *STM* impinge on multiple hormone pathways to regulate SAM development and function.

The organ-boundary-associated genes *CUP-SHAPED COTYLEDON1* (*CUC1*), *CUC*2 and *CUC3* are required for activation of *STM* expression and the subsequent formation of the SAM during embryogenesis (Aida et al., 1999; Takada et al., 2001; Hibara et al., 2003; Vroemen et al., 2003). They also function in the specification of meristem-organ boundary zones, with combinatorial loss-of-function mutants displaying fusion of the cotyledons in addition to loss of SAM formation. *CUC1* is initially expressed throughout the embryonic SAM but becomes restricted to the boundary zones following activation of *STM* expression (Takada et al., 2001). A previous study has revealed that STM binds the *CUC1* promoter and directly promotes *CUC1* expression, suggesting that STM and CUC1 comprise a positive transcriptional feedback loop (Spinelli et al., 2011). However, the different expression patterns of *STM* and *CUC1* suggest that this feedback loop must be attenuated by additional factors in order to resolve CUC1 to the meristem-organ boundary.

Using inducible regulation of *STM* and differential expression analysis, we have identified STM-responsive genes and have coupled this with Bayesian network inference based on publicly available microarray data to infer the structure of the STM GRN. We reveal regulatory associations between *STM* and key TFs involved in multiple aspects of SAM function, including organ formation and differentiation, regulation of phyllotaxis and the establishment of meristem-organ boundary zones. We find that a direct positive-feedback loop exists between STM and CUC1 attenuated by STM regulation of the CUC1-targeting *miR164c*. *In silico* modelling shows that, together with STM protein movement, this can potentially explain *STM* and *CUC1* expression patterns in the SAM and meristem-organ boundary, respectively.

## RESULTS

### Identification of STM-responsive genes using STMoe timecourse analysis

**Fig. 1.**
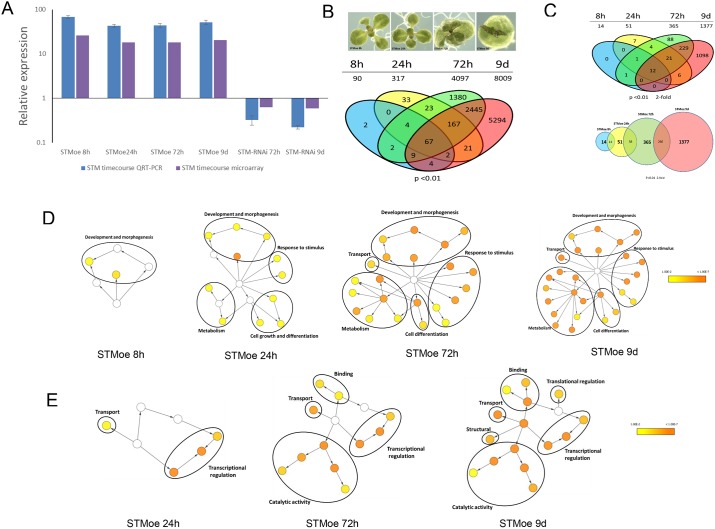
**Transcriptomic analysis of timecourse data.** (A) *STM* expression (mRNA levels following induction with DEX. For each time-point, the STMoe line (S3.8) was compared with an empty-vector control line (23.7) under identical induction conditions. Purple bars indicate STM mRNA levels, as measured on an Affymetrix ATH1 microarray using RMA/LIMMA with *P*<0.01. Blue bars indicate STM mRNA levels, as measured by QRT-PCR. Error bars indicate s.d. Three biological replicates were used for each time-point. (B) Morphology of STMoe timecourse seedlings and comparison of DEGs to identify exclusive overlaps at each time-point. A *P*-value cut-off of <0.01 was used to identify DEGs. Total number of DEGs is shown below each time-point. (C) (Top) DEG overlaps at each time-point using *P*<0.01 and a minimum 2-fold expression change filter. (Bottom) Comparison of DEGs to identify overlaps at successive time-points. Numbers in bold represent total number of DEGs with *P*<0.01 and a minimum 2-fold expression change at the respective time-point. Genes showing reciprocal expression dynamics between time-points were omitted. (D,E) Gene ontology (GO) SLIM enrichment analysis of DEGs identified at each STMoe time-point. All nodes were set to the same size for clarity. Progressive enrichment of GO categories is evident across the timecourse for biological process (D) and molecular function (E). No significant enrichment was found for molecular function at the STMoe 8 h time-point. Enriched GO categories are circled and given a generalised annotation term for simplicity. The full list of enriched GO categories is shown in Fig. S1.

Given the central importance of STM in SAM development and function, we identified components of the STM GRN using differential expression analysis of timecourse data for steroid-inducible *STM* upregulation and downregulation by RNAi, achieved using the dexamethasone-inducible TGV system (Bohner et al., 1999; Scofield et al., 2013). STM overexpression (STMoe) was induced for 8 h, 24 h, 72 h or 9 days prior to harvesting and RNA isolation from aerial tissue. To complement this approach, we induced downregulation of STM (STM-RNAi) for 72 h or 9 days. All plants were harvested 9 days after sowing (DAS) and RNA samples were analysed using DNA microarrays. Fig. 1A shows the quantification of *STM* transcript levels in the STMOE and STMRNAi timecourse (purple bars). *STM* transcript levels measured in the same samples by real-time quantitative RT-PCR (qRT-PCR) showed greater response in both STMoe and STM-RNAi samples compared with microarray quantification, suggesting that the microarray-derived expression values are conservative (Fig. 1A, blue bars), at least for the *STM* probesets.

We identified differentially expressed genes (DEGs) in each sample (using RMA and LIMMA) and compared the overlap between STMoe datasets (Fig. 1B,C). As expected, the number of DEGs increased progressively from STMoe 8 h (90 with no fold-change filter; 14 with 2-fold change filter) to the STMoe 9 days sample (8009 with no fold-change filter; 1377 with 2-fold change filter; Table S2). The majority (>70%) of DEGs identified at a given time-point were also differentially expressed at later time-points, indicating that these genes were displaying a robust and consistent change in expression dynamics.

The progressive expansion of numbers of DEGs is also reflected in the incremental expansion of over-represented Gene Ontology (GO) categories at each successive time point (Fig. 1D,E; Fig. S1; Table S1), with biological process terms in the STMoe 8 h time-point showing enrichment of genes involved in ‘development and morphogenesis’, expanding to include ‘cell differentiation’, ‘metabolism’, ‘transport’ and ‘response to stimulus’ at later time-points. For GO molecular function, no enriched terms were identified at STMoe 8 h, but ‘transcriptional regulation’ and ‘transport’ were enriched at STMoe 24 h, expanding at later time-points to include ‘catalytic activity’ and ‘binding’.

### Identification of STM-responsive genes using meta-analysis

The above approach led to the identification of DEGs that might comprise directly regulated STM target genes, indirectly-regulated genes or changes in gene expression arising from secondary effects associated with the altered morphology of 72 h and 9 day samples (Fig. 1B). Therefore, to robustly identify the most biologically relevant DEGs likely to be directly regulated by STM, we performed a meta-analysis (combining *P*-values using Fisher's inverse chi-squared test) of DEGs in the early STMoe (STMoe 8 h and STMoe 24 h) and STMRNAi (72 h and 9 days) datasets. Despite showing clear downregulation of STM transcript levels, the STMRNAi datasets were limited in power and sensitivity due to the conservative microarray expression level fold-changes, preventing straightforward direct comparison with STMoe datasets to identify genes showing reciprocal regulation. Hence, combining these datasets with the early STMoe time-point data provided additional power to capture biologically relevant DEGs.

**Fig. 2.**
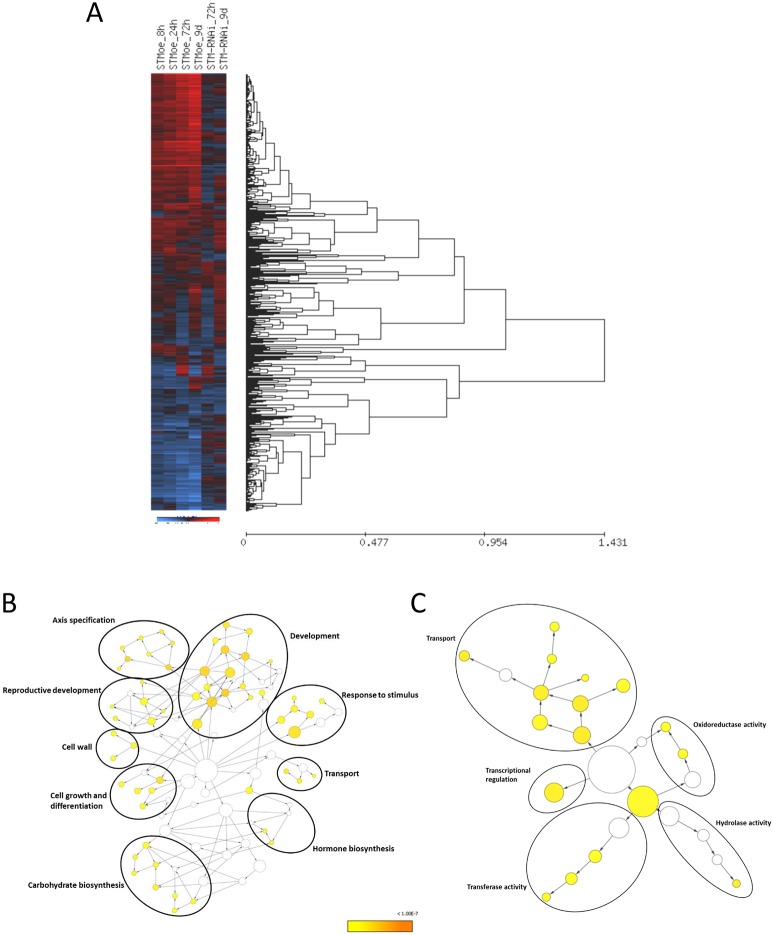
**Meta-analysis of timecourse data.** (A) Hierarchical clustering (average distance UPGMA) of 465 meta-analysis DEGs in STMoe and STM-RNAi timecourse. Red indicates an increase in gene expression, blue indicates a decrease in gene expression. (B,C) GO (full) enrichment analysis for 465 DEGs identified in the meta-analysis for biological process (B) and molecular function (C). Circled areas encompass nodes corresponding to related biological processes/molecular functions, and are assigned a generalised annotation. Full GO annotations are shown in Fig. S2.

Meta-analysis of STMoe 8 h, STMoe 24 h and STM-RNAi datasets using Fisher's inverse chi-squared test identified 465 DEGs with an FDR-corrected q-value significance of <0.01 (Fig. 2; Table S4). Hierarchical clustering of expression profiles of these DEGs across the timecourse (Fig. 2A) revealed some genes showing consistent upregulation or downregulation across the timecourse in response to STM, whereas others showed more-complex expression dynamics.

Gene Ontology (GO) analysis for biological processes revealed enrichment of genes associated with multiple aspects of meristem function including ‘hormone regulation’ (auxin biosynthesis and transport, cytokinin signalling, GA biosynthesis), as established in previous studies, ‘cell wall modification’ and ‘carbohydrate biosynthesis’ (cellulose, callose, lignin and xyloglucan modifying enzymes), ‘control of cell growth and differentiation’ (cyclins, differentiation-associated TFs), ‘tissue morphogenesis’ and ‘development’ (mostly TFs involved in shoot, organ and reproductive development), and ‘axis/polarity specification’, ‘transport’ and ‘response to biotic/abiotic stimuli’ (Fig. 2B; Fig. S2; Table S1). For molecular function, enriched terms included ‘transcriptional regulation’, ‘transport’, ‘oxidoreductase-, hydrolase- and transferase activity’ (Fig. 2C). These enriched GO terms are similar to those revealed through analysis of the individual time-points in the timecourse data, demonstrating that the meta- and timecourse analyses were consistent in the identification of genes with common functions.

### Bayesian network analysis reveals STM GRN topology

**Fig. 3.**
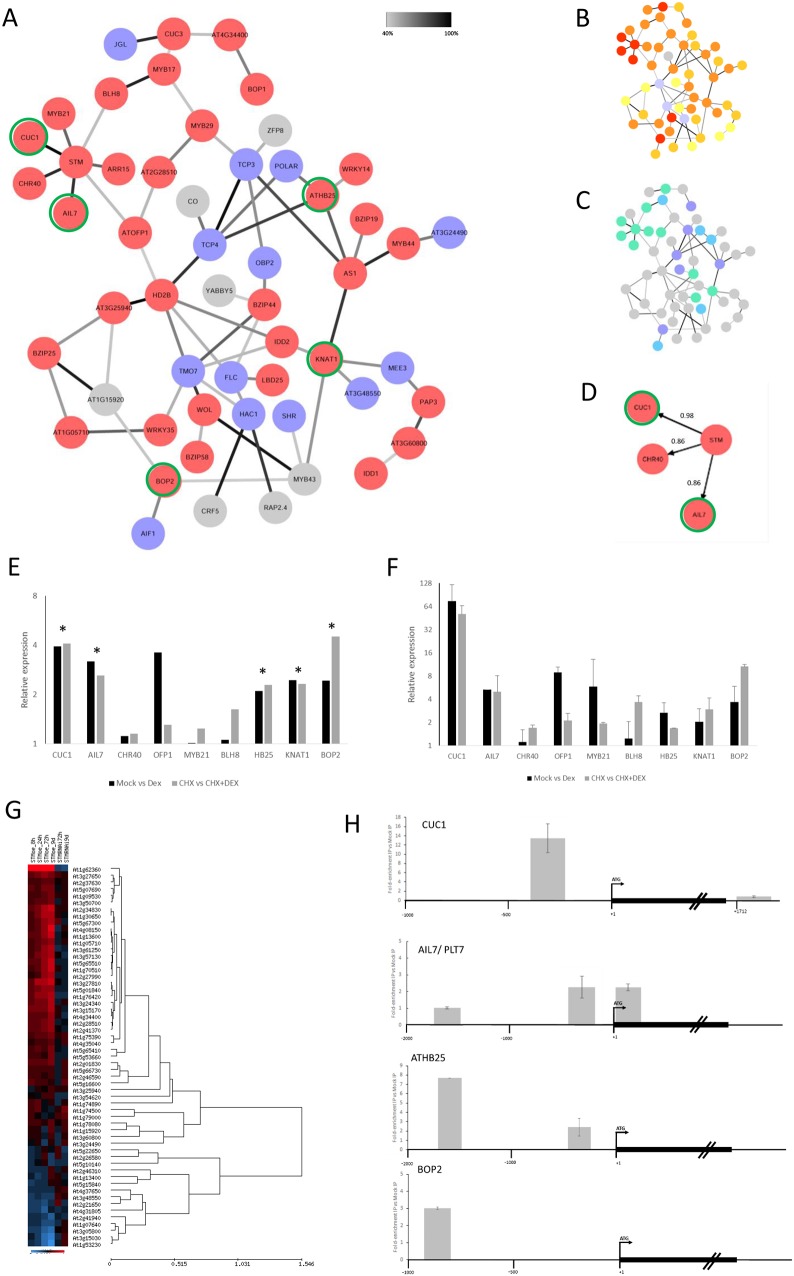
**STM Bayesian network analysis.** (A) Consensus Bayesian network analysis of STM-responsive TFs identified by meta-analysis. Nodes in red indicate genes showing upregulation in response to increased STM activity, blue nodes indicate downregulation. Grey nodes indicate genes with variable differential expression across the timecourse. Darker lines (edges) indicate frequency of prediction: 40% (light grey) to 100% (black). Edge significance is the sum of both possible directions. Green outer circles indicate potential direct STM target as based on microarray data (see Materials and Methods). Fifty-four out of 57 transcription-associated genes show inter-connections (>40%) with the rest of the network. (B) Consensus Bayesian network analysis showing earliest response times in STMoe timecourse. Red, STMoe 8 h; dark orange, STMoe 24 h; light orange, STMoe 72 h; yellow, STMoe 9 days; blue, STM-RNAi 72 h or 9 days. (C) Expression patterns based on re-analysis of the data from Yadav et al. (2009). Green, expressed specifically in SAM; purple, expressed specifically in organ primordia; blue, expressed in SAM and organ primordia, either pWUS and pFIL domains (corpus) or pCLV3 and pFIL domains (tunica); grey, general non tissue-specific expression. (D) Local network of STM and the three most high-confidence associations. Arrows indicate that STM acts as a parent node to CUC1, AIL7 and CHR40. (E) Quantification of mRNA levels of key network genes following 3 h induction of STM-GR fusion protein with 60 µm DEX compared with mock-induced sample (black bars) or DEX+CHX compared with CHX-treated sample (grey bars) using Affymetrix ATH1 DNA microarrays. Three biological replicates were performed. Asterisks indicate significant (*P*<0.01) and >2-fold expression changes. (F) Quantification of mRNA levels as in E using qRT-PCR. Three biological replicates were performed. (G) Hierarchical clustering (average distance UPGMA) of 57 meta-analysis DEGs encoding mostly transcriptional regulators in STMoe and STM-RNAi timecourse. Red indicates an increase in gene expression, blue indicates a decrease in gene expression. (H) ChIP analysis of STM-GR binding to gene promoter sequences. Gene promoters are shown schematically, with amplicons shown as grey boxes. IP samples were compared with mock-IP samples. Relative enrichment of fragments by qRT-PCR is shown on overlying graphs compared with control *ACTIN2* amplicon. At least three biological replicates were performed for *CUC1* and *AIL7*. Two biological replicates were performed for AtHB25 and BOP2. All error bars indicate s.d.

GO analysis revealed that genes encoding transcriptional factors (TFs) were enriched among the STM-responsive genes, indicating that STM may function largely through controlling the expression of other transcriptional regulators. To explore the potential regulatory interactions among the STM-associated transcriptional regulators, we generated a Bayesian network (Heckerman and Wellman, 1995; Friedman, 2004) using discretised relative gene expression data from over 2000 publicly available transcriptomic microarray datasets. This enabled a preliminary GRN to be constructed comprising mostly TFs and other factors identified in the meta-analysis that impinge primarily on transcriptional regulation. A total of 57 genes were used for network construction (Fig. 3G; Table S5). Bayesian analysis predicted the frequency of conditional dependency relationships, indicated as edges between nodes (in both directions), and different frequency threshold cut-offs were explored to generate maximum network connectivity, selecting >40% confidence as the optimum threshold. Of the 57 genes used for network construction, 54 displayed conditional dependency relationships in the Bayesian network with >40% confidence and were used to generate a consensus network (Fig. 3A, shown without directionality for clarity; Fig. S3, with directionality).

We surmised that genes for which STM was assigned as a direct parent (connected by a single edge) are those most likely to represent direct STM targets. In agreement with this, they generally displayed the earliest response to increased STM levels in the STMoe timecourse data (Fig. 3B; Fig. 3G; Fig. S4). We analysed the spatial expression domains of network genes using the high-resolution dataset from Yadav et al. (2009). This revealed that several putative direct STM target genes are expressed in the SAM, whereas genes in more-distal branches of the network were more likely to show organ primordium-associated expression (Fig. 3C). Of all the genes in the network, the transcription factors *CUC1* and *AIL7/PLT7*, and the SNF2 chromatin remodelling factor *CHR40* showed the strongest connection to *STM*, with *STM* being assigned as the parent node of these genes with confidences of 98%, 86% and 86%, respectively (Fig. 3D; full directional Bayesian network Fig. S3). These genes were positively regulated in the timecourse (red nodes; Fig. 3A), whereas genes that function in the promotion of leaf differentiation, such as *TCP3* and *TCP4*, showed down-regulation in response to STM (blue nodes; Fig. 3A). Numerous other TFs with known roles in SAM function were also involved in the network, including the KNOX gene *KNAT1/BP*, the BEL1-like homeobox gene *BLH8/PNF*, the boundary-associated genes *CUC3* and *LBD25*, and differentiation-associated factors such as *AS1*, *BOP1* and *BOP2*. Genes encoding TFs involved in hormone regulation or response were also identified, including *ARR15*, *HB25*, *OFP1*, *CRF5*, *ZFP8* and *MYB21*. The responses of a subset of network components to STM were confirmed by qRT-PCR in separate experiments (Fig. S5).

To determine whether regulation of genes in the network by STM was direct, we made use of non-transcriptional induction of STM using a p35S::STM-GR fusion protein line (Brand et al., 2002), comparing gene expression responses when STM is induced with DEX in the absence and presence of the protein-synthesis inhibitor cycloheximide (CHX). Genes responding in the presence of CHX do not require protein synthesis and are therefore likely direct STM targets. Using microarray analysis, we identified several genes from the network showing significant differential expression (*P*<0.01) and a minimum 2-fold change in expression level in both mock versus DEX treatments and CHX versus CHX+DEX treated samples (Fig. 3E,F; *CUC1*, *AIL7/PLT7*, *HB25*, *KNAT1/BP* and *BOP2*). These constitute candidate directly-regulated STM target genes.

To test whether STM binds directly to the promoters of these putative direct targets, we tested a subset by chromatin immunoprecipitation (ChIP) of DEX-treated 35S:STM-GR samples using an anti-GR antibody to determine whether enrichment of target gene promoter sequences was detected by qRT-PCR in immunoprecipitated (IP) versus a mock-IP control. This revealed that *CUC1*, *BOP2*, *HB25* and *AIL7/PLT7* have promoter sequences directly bound by STM (Fig. 3H), with CUC1 showing the highest level of enrichment of sequences predicted to bind STM *in silico*. This is in agreement with previous work demonstrating that *CUC1* is a direct STM target (Spinelli et al., 2011).

### STM and CUC1 form a direct positive-feedback loop attenuated by miR164c

**Fig. 4.**
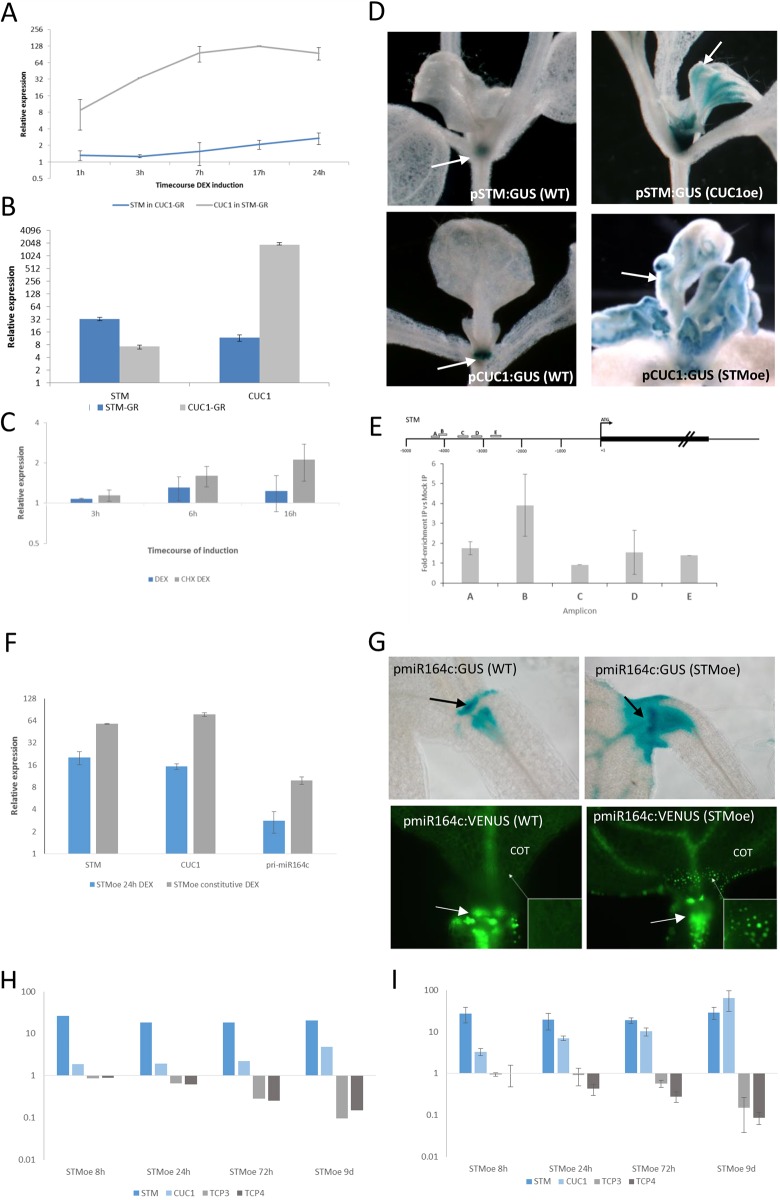
**Regulatory interactions among STM, CUC1, TCPs and miR164c.** (A) qRT-PCR timecourse analysis of *STM* and *CUC1* expression levels in DEX-induced CUC1-GR and STM-GR lines relative to mock-induced control lines. (B) qRT-PCR analysis of *STM* and *CUC1* expression levels in long-term induced STM-GR and CUC1-GR lines relative to wild-type control lines. (C) qRT-PCR analysis of *STM* expression levels in response to induction of CUC1-GR with DEX versus mock induction or with cycloheximide (CHX) and DEX versus CHX. CHX-DEX treatment was discontinued after 16 h owing to toxicity effects. (D) pSTM:GUS (top) and pCUC1:GUS (bottom) activity (blue) in wild-type and STMoe or CUC1-GR (CUC1oe) seedlings treated with DEX. Arrows indicate GUS activity. (E) ChIP analysis of the upstream region (promoter) of *STM*. (Top) Schematic representation of positions of tested amplicons upstream of START codon. (Bottom) Quantification of ChIP amplicons in the IP sample relative to mock-IP sample. Results are based on two biological replicates. (F) qRT-PCR analysis of STM, CUC1 and pri-miR164c expression in STMoe samples 24 h after induction with DEX (STMoe 24 h DEX) and in plants treated with DEX from germination (STMoe constitutive DEX). (G) Expression of pmiR164c:GUS (top) and pmiR164c:VENUS (bottom) in wild-type and STMoe seedlings (arrow=SAM) treated with DEX. A pmiR164c:VENUS reporter is expressed ectopically in cotyledon bases in STMoe. Arrows indicate GUS or GFP activity. (H,I) Expression levels of *STM*, *CUC1*, *TCP3* and *TCP4* in STMoe timecourse measured by ATH1 DNA microarray (H) and by qRT-PCR (I) in separate experiments. All experiments are based on at least three biological replicates unless otherwise stated. Error bars indicate s.d. All expression changes in H are significant (*P*<0.01) except for *TCP3* and *TCP4* in the STMoe 8 h sample.

Genetic analysis has shown that CUC1/CUC2 function is required for activation of *STM* expression during embryonic development (Takada et al., 2001), whereas STM has been shown to directly regulate *CUC1* expression (Spinelli et al., 2011). Here, we have identified *CUC1* as an early responding target of STM directly connected to STM in the Bayesian network where it has the highest conditional dependency on STM of all network components. We further confirmed that it is a direct STM target *in vivo* using both induction in the presence of CHX and ChIP. Together, these data suggested the possibility that STM and CUC1 could have a mutually reinforcing regulatory relationship, and potentially even direct positive feedback at the transcriptional level. To determine whether CUC1 transcriptionally activates *STM* in adult plants and, if so, to determine the relative activation potential of STM for CUC1, and vice versa, we measured *STM* and *CUC1* transcript levels using qRT-PCR in a timecourse using DEX-inducible pRPS5a:mCUC1-GR (CUC1-GR) and p35S:STM-GR (STM-GR) lines, respectively. We found that *STM* indeed displayed a response to CUC1 induction, but this was slower and less dramatic than the rapid and robust response of *CUC1* to STM induction (Fig. 4A). *STM* and *CUC1* levels were also elevated in long-term induced, phenotypic CUC1-GR and STM-GR lines, respectively, as determined by both qRT-PCR and promoter:GUS reporter gene analysis (Fig. 4B,D). Furthermore, we found that *STM* responded to CUC1-GR even in the presence of CHX (Fig. 4C), suggesting direct regulation. The direct regulation of STM by CUC1-GR was confirmed by ChIP, which showed that CUC1 binds to a region ∼4 kb upstream of the *STM* initiation codon (Fig. 4E). We conclude that CUC1 directly regulates *STM* expression, and that, taken together, these data show that the STM and CUC1 transcription factors could promote one another's expression in a direct positive-feedback loop.

**Fig. 5.**
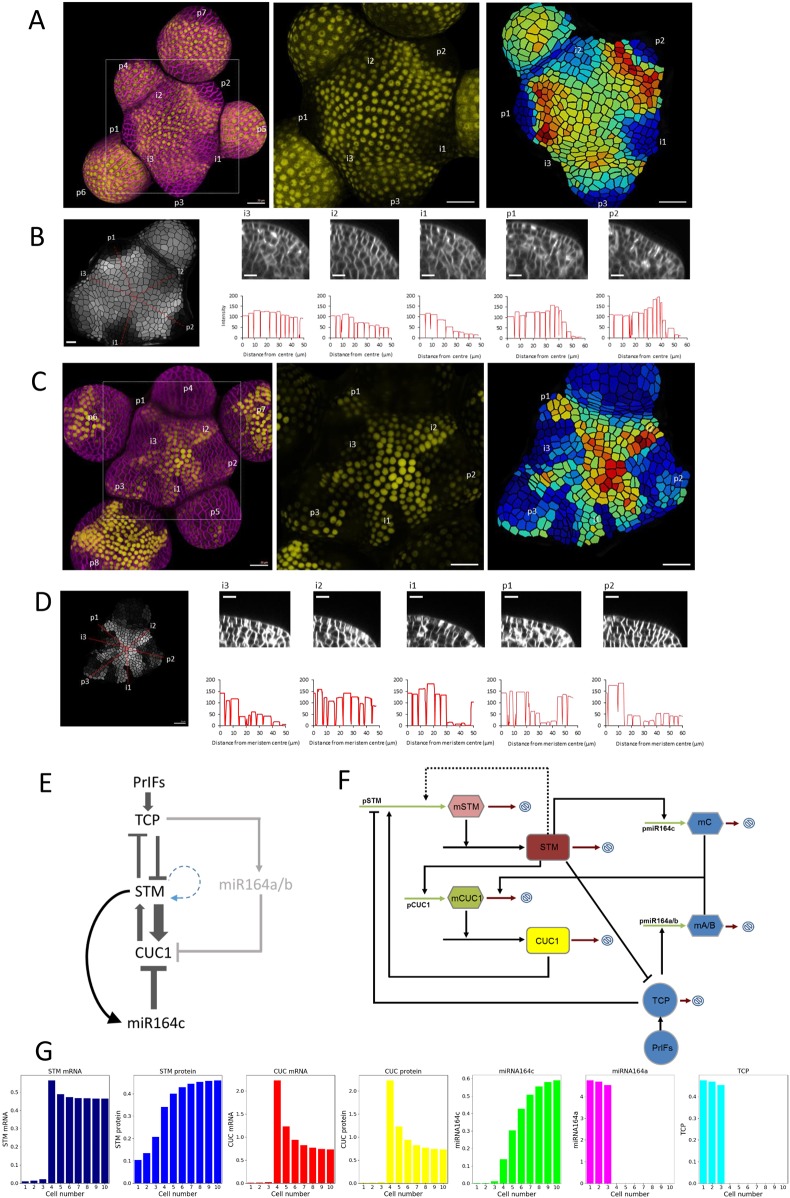
**Imaging and modelling of STM-CUC1-TCP-miR164c expression dynamics and interactions.** (A) Quantification of pSTM::STM-VENUS signal in the inflorescence meristem. (Left) 2.5D projection of meristem layer 1 produced from a confocal stack. VENUS signal is in yellow. FM4-64-stained cell membranes are in magenta. Developing primordia (p) and incipient primordia (i) are indicated. p1 is the first primordium at which outgrowth was detected in cross section. Organ sequence was inferred from organ size and the expected phyllotactic pattern. (Middle) Total VENUS fluorescence in layer one of the SAM. VENUS signal is absent in i1, p1, p2 and p3, and reduced in the vicinity of i2, but not i3. (Right) Segmented image showing cellular compartments extracted from the FM4-64 channel. Shading indicates total VENUS signal per cell. Blue indicates low signal intensity, red indicates high signal intensity. A gradient of VENUS signal intensity is detected at the boundary of i1 and i2 in comparison with the sharper boundaries of older primordia. Scale bars: 20 µm. (B) Quantification of pSTM::STM-VENUS signal intensity across the meristem-organ boundary. (Left) Greyscale image of a segmented SAM, showing total VENUS signal intensity per cell. Red lines indicate transects across the meristem-organ boundary of primordia of different ages. (Right, top) Cross-sections through the SAM corresponding to transects indicated in left image. Scale bars: 10 µm. (Right, bottom) Quantification of VENUS signal in transects. Troughs indicate the cell boundaries. A gradient of STM activity is formed between i3 and i1 before upregulation of STM in the boundary in p1 and p2. Scale bars: 10 µm. (C) Quantification of pmiR164c::VENUS signal in the inflorescence meristem performed as described in A. Scale bars: 20 µm. (D) Quantification of pmiR164c::VENUS signal intensity across the meristem-organ boundary performed as described in B. Scale bars: 20 µm (left image) and 10 µm (right top transect images). pmiR164c::VENUS signal is absent within primordia and there is a sharp gradient of signal at the meristem-organ boundary. (E) Schematic representation of STM, CUC1, TCP and miRNA164c interactions based on data in this study and previous studies. PrIFs, primordium identity factors, e.g. auxin, that promote TCP expression. Potential autoregulation of *STM* is indicated with a dashed blue curved arrow. Promotion of miR164a/b expression by TCP (Koyama et al., 2010) is shown with a grey arrow. (F) Schematic representation of model interactions. Promoters, pSTM, pCUC1, pmiR164c and pmiR164a/b; mRNAs, mSTM and mCUC1; microRNAs, mA/B and mC; proteins, STM, CUC1 and TCP. Arrows followed by strike-out circles indicate degradation. (G) Steady-state model outputs incorporating *STM* autoregulation. Cells 1-3 correspond to the primordium; cells 4-10 correspond to more centrally located cells in the meristem.

Such reciprocal positive regulation of *STM* and *CUC1* would be paradoxical as these genes have strikingly different expression patterns. *STM* is broadly expressed throughout the SAM but excluded from incipient organ primordia (Long et al., 1996; Fig. 5A), whereas CUC1 protein is confined to meristem-organ boundary zones, as seen with pCUC1:CUC1-GUS and pCUC1:CUC1-GFP fusion proteins (Fig. S5; Sieber et al., 2007). Regulation of *CUC1* mRNA by microRNAs of the miR164 subfamily restricts its localisation, as shown by the broader pattern observed with a miR164-resistant fusion protein reporter pCUC1:mCUC1-GFP (Sieber et al., 2007; Fig. S5) and activity of the pCUC1:GUS reporter construct throughout the SAM (Fig. S5). A previous study has suggested that STM promotes expression of *miR164a/b* (Spinelli et al., 2011), but miR164a/b are expressed in leaf primordia under the control of TCP transcription factors, suggesting that they are not normally involved in *CUC* regulation within the SAM. In contrast, expression of *miR164c* is reported to be localised to the SAM in a similar pattern to *STM* (Sieber et al., 2007; Fig. 5C). We therefore quantified expression of *STM* and *miR164c* across the SAM using pSTM:STM-VENUS and pmiR164c:VENUS reporters, respectively.

pSTM:STM-VENUS was consistently excluded from primordia from stage i2 onwards (*n*=8). Quantification of pSTM:STM-VENUS signal revealed a gradient of expression between the SAM centre (high) and incipient primordia (low; Fig. 5A,B) where expression of the synthetic auxin reporter DR5:VENUS was detected (Fig. S5). In older primordia displaying outgrowth, we saw *STM* expression increased at the meristem-organ boundary, possibly due to mechanical forces (Landrein et al., 2015) or symplastic isolation between primordium and meristem (Bayer et al., 2008) together with transactivation by CUC1 after boundary establishment.

Quantification of pmiR164c:VENUS revealed expression throughout the SAM (Fig. 5C,D). The expression pattern of pmiR164c::VENUS showed greater variability than that of pSTM:STM-VENUS; however, expression was consistently strongest in the centre of the meristem and lower in the peripheral zone (*n*=10, Fig. S7). Although pmiR164c:VENUS was not completely removed from outgrowing primordia, most i1 incipient primordia showed reduced pmiR164c:VENUS expression compared with the central region (observed in 7/10 stems). Similarities between the expression patterns of pSTM:STM-VENUS and pmiR164c:VENUS suggest that transcription of miR164c may be, at least in part, regulated by STM. We therefore measured levels of the pre-microRNA pri-miR164c before its processing into mature miRNA and found that levels increased following induction of STM (Fig. 4F), suggesting that STM can activate the expression of miR164c, as further supported by ectopic miR164c expression observed in STMoe plants using pmiR164c:GUS and pmiR164c:VENUS reporters (Fig. 4G). Together, these data indicate that miR164c transcription is upregulated by STM.

Our microarray analysis also showed that STM represses the expression of *TCP3* and *TCP4*, key factors in the specification of organ primordia, leaf differentiation and repression of KNOX gene expression (Li, 2015), and we confirmed this by qRT-PCR (Fig. 4H,I; Fig. S5). *TCP3* and *TCP4* showed a gradual decline in transcript levels across the *STM* induction timecourse, with levels being most greatly reduced at the STMoe 9 days time-point. However, the relatively slow repression coupled with the lack of direct connections to STM in the network suggests indirect repression by STM. The repression of TCP gene expression by STM provides a potential mechanism for preventing precocious organ formation or differentiation in the SAM. Coupled with the known repression of KNOX gene expression by TCPs (Li et al., 2012; Aguilar-Martínez and Sinha, 2013), this suggests a mutually antagonistic relationship that leads to mutual inhibition of pluripotent and differentiated cell fates.

### A model for localisation of CUC1 to organ boundaries

The data presented above suggest a model involving mutually antagonistic regulation between STM and TCPs, leading to exclusion from each other's expression domains, together with a mutually promoting regulatory module between STM and CUC1 composed of a direct positive-feedback loop attenuated by *miR164c* (Fig. 5E). The outcome of these interactions is not straightforward to predict in a spatial context, particularly as intercellular movement of STM occurs and is required for its function (Kim et al., 2003; Balkunde et al., 2017).

We sought to understand whether the simple rules imposed by mutual exclusion and the positive-feedback loop could explain the establishment of observed *STM* and *CUC1* gene expression patterns within the SAM and incipient meristem-organ boundaries, respectively. We therefore created an ordinary differential equation (ODE)-based model to simulate the regulatory interactions between STM, CUC1 and miR164c, together with primordium identity factors (PrIFs) that specify primordium identity and promote expression of TCPs, which repress STM expression in primordia and are, in turn, repressed by STM in the meristem (Fig. 5E,F; Appendix S1). TCPs also promote expression miR164a/b, which targets *CUC1* transcripts for degradation in organ primordia. PrIFs represent factors such as auxin, which are crucial in the establishment of organ primordium identity (Hay et al., 2006), although the mechanistic details of primordium establishment are beyond the scope of our model.

In this model, a cross-section of the SAM is represented by a one-dimensional file of 10 cells, with one end (left) corresponding to an incipient organ primordium and the other (right) to central SAM cells (Fig. 5G). The model is initiated with uniform STM activity across the meristem and organ primordium identity defined by PrIFs in three cells at the end(s) of the cell file. We then numerically simulated the evolution of the model until the levels of components in each cell in the file reached a steady state. Remarkably, these simple interactions recapitulated the observed accumulation of CUC1 at the meristem-organ boundary (Fig. S6; Fig. 5G) using the parameters listed in Fig. S8, identified through parameter exploration guided by data from this and other studies (Narsai et al., 2007). Local parameter sensitivity analysis was performed to assess the dependence of simulation outputs on parameter values (Appendix S1).

In organ primordia, the model reflects that TCPs transcriptionally repress STM and upregulate miR164a/b, preventing accumulation of *CUC1* transcripts. In the SAM, STM-CUC1 positive feedback is predicted to create a region of relatively high *STM* and *CUC1* expression, and diffusion of STM along the file generates an instructional gradient of reducing STM activity from central zone (CZ) to primordium, in agreement with imaging data (Fig. 5A-D). As STM levels are higher in the CZ than at the organ boundary at this early stage following primordium initiation, the expression of *miR164c* is also highest in the CZ and declines towards the primordium. The observed high sensitivity of *CUC1* to STM levels creates relatively high CUC1 mRNA expression in both the CZ and organ boundary, despite the variation in STM levels. The gradient of *miR164c* expression that arises from its quantitative response to STM leads to the exclusion of *CUC1* transcripts from the CZ; however, in the organ boundary zone, relatively low levels of *miR164c* are insufficient to degrade CUC1 mRNA, leading to accumulation of CUC1 protein (Fig. 5G).

As STM is found throughout the SAM, its expression is likely not solely dependent on CUC1. Autoregulation has been demonstrated for orthologous KNOX genes in rice (Tsuda et al., 2011). In agreement with this, we observed that the pSTM:GUS reporter is ectopically expressed in STMoe plants. We therefore included STM autoregulation (when compared with basal STM expression) in our model and found that this further enhanced recapitulation of published expression patterns (Fig. 5G, Figs S5 and S6). Interestingly, and consistent with recent findings of the essential requirement of STM mobility for its function (Balkunde et al., 2017), we found that diffusion of STM was crucial for STM gradient formation and hence the proper resolution of CUC1 expression at the meristem-organ boundary (Fig. S6).

## DISCUSSION

The essential role for *STM* in the establishment and maintenance of the SAM has been described in numerous studies (Long et al., 1996; Clark et al., 1996; Scofield et al., 2013), and KNOX genes have been implicated in the repression of GA biosynthesis and signalling and activation of CK biosynthesis (Sakamoto et al., 2001; Jasinski et al., 2005; Yanai et al., 2005). Others have suggested a role for KNOX genes in the control of cell wall modification via repression of lignification (Mele et al., 2003). Recent genome-wide approaches to identify KNOX-regulated target genes and associated biological processes of the orthologous KN1 gene in maize (*KN1*) revealed a further strong association with auxin-associated factors (Bolduc et al., 2012), and in rice highlighted the importance of brassinosteroid (BR) catabolic genes in the ortholog *OSH1* GRN (Tsuda et al., 2014).

Here, we define genes that are transcriptionally responsive to STM using STMoe and STM-RNAi timecourse data and meta-analysis. We identify several genes involved in auxin biosynthesis and transport, and in GA biosynthesis, in addition to genes involved in CK catabolism, signalling and response, with additional genes in these hormone pathways showing differential expression in the STMoe 9 days dataset (Table S6). We also identified several genes encoding proteins involved in cell wall modification, such as expansins, enzymes involved in lignification, cellulose synthases, xyloglucan-modifying enzymes and callose synthases. Hence, our data support the conclusions made in other studies that KNOX genes regulate hormone pathways and cell wall modification, functions that are reflective of its important developmental role.

Genes encoding transcription factors were enriched (over-represented) in the meta-analysis dataset. This suggests a high-order regulatory role for STM in coordinating the expression of many developmental transcription factors, which themselves in turn regulate different aspects of meristem function. Using the subset of these genes that represent primarily transcription factors as potential components of the STM GRN, we performed Bayesian conditional dependency analysis to infer parent-child relationships and constructed a consensus GRN. This used discretised data from over 2300 publicly available *Arabidopsis* Affymetrix microarray datasets defined only by the chosen experimental descriptors of ‘seedling’ and ‘shoot apex’, which were discretised as to whether each gene was overexpressed, under-expressed or unchanged relative to the normalised average across all the arrays. Hence, although the network components were identified by the timecourse microarray analysis presented here, the conditional dependency relationships used to infer network structure/topology were obtained using independent experimental data from a large number of experiments in many labs.

In plants, GRNs for specific responses such as pathogen infection have been derived using dynamic Bayesian approaches from fine-grained timecourses from equivalent samples (Penfold et al., 2012). However, there are limited studies using numerous independent microarrays to generate GRNs using Bayesian approaches (reviewed by Banf and Rhee, 2017), e.g. Needham et al. (2009), who inferred regulatory interactions from equivalent microarray datasets using an alternative approach of growing a network derived for a small initial set of genes by iterative addition of genes to produce the optimal network structure.

In this study, our approach resulted in informative networks reflecting that many of the genes that responded to STM at the earliest time-points were directly connected to STM by a single edge in the network, inferring a direct parent-child relationship and suggesting that this conditional dependency analysis captures potential regulatory relationships. This is further supported by the finding that most of the genes directly connected to STM are expressed in the SAM (Yadav et al., 2009), and two of the three genes most strongly connected to STM (*CUC1* and *AIL7/PLT7*) were shown to be direct targets using ChIP and CHX experiments.

Indeed, several genes previously shown to be functionally or physically associated with STM were located close to STM in the network. The BEL1-like protein BLH8/POUNDFOOLISH (PNF), a known binding partner of KNOX1 proteins (Kanrar et al., 2006), the A-type cytokinin response regulator ARR15 and the ovate family protein OFP1, which interacts with KNOX proteins to repress GA synthesis (Hackbusch et al., 2005; Wang et al., 2007), were all directly connected to STM in the network with varying degrees of confidence. However, our analysis did not identify these as direct STM targets and not all genes shown to be direct STM targets were closely associated with STM in the network. *BOP2*, which is involved in KNOX gene repression in leaves (Ha et al., 2010), *ATHB25*, which is involved in GA synthesis (Bueso et al., 2014), and *KNAT1/BP* were all connected to STM by at least four edges. Nevertheless, the network analysis recapitulated several previously described relationships, such as regulation of *CUC1* by STM (Spinelli et al., 2011) and of *KNAT1/BP* by AS1, suggesting that this approach makes useful predictions as a basis for experimentation.

Our analysis revealed several novel associations between STM and other developmental transcriptional regulators. Notably, we identified a direct connection between *STM* and *AIL7/PLT7*, a member of the PLETHORA family of AP2 domain transcription factors, and show it is likely to be a direct STM target. *AIL7/PLT7*, together with related *AIL6/PLT3* and *AIL5/PLT5*, is required for proper phyllotaxis, the repression of differentiation and promotion of cell division in the SAM (Prasad et al., 2011; Mudunkothge and Krizek, 2012; Kareem et al., 2015). Given that loss of STM function leads to excessive SAM cell differentiation and defects in phyllotaxis (Long et al., 1996; Clark et al., 1996), it is plausible that STM controls these processes, at least in part, through regulation of *AIL7/PLT7*, and potentially also through *AIL6/PLT3*, which showed upregulation at later STMoe time-points (Table S7).

We identified TCP3 and TCP4 in our meta-analysis and showed that these genes are downregulated in response to STM. Using data from the STMoe 9 days time-point, we also observe STM-dependent repression of other class 2 TCPs, including *TCP2*, *TCP5*, *TCP10*, *TCP17* and *TCP24* (Table S7), suggesting that such repression is common among the majority of class-2 TCP family members. Class-2 TCPs have been shown to promote leaf differentiation and antagonise shoot development and function (Palatnik et al., 2003; Koyama et al., 2007, 2010; Sarvepalli and Nath, 2011; Schommer et al., 2014), and interact with the LOB-domain factor AS2 in the direct repression of KNOX gene (*KNAT1/BP* and *KNAT2*) expression (Li et al., 2012). In addition, some members of the class 1 TCP gene family have also been shown to transcriptionally repress KNOX genes *STM* and *KNAT1/BP* (Aguilar-Martínez and Sinha, 2013). Hence, our data show that the repression of KNOX gene expression by TCPs is a reciprocal relationship, with KNOX genes also acting to repress TCP expression (especially class 2 genes), thereby excluding their differentiation-promoting function from the SAM.

*CUC1* showed the strongest association with *STM* in the Bayesian network. As *CUC1* is bound and activated by STM (here and Spinelli et al., 2011) and CUC1 binds and activates *STM*, these two transcription factors comprise a direct positive-feedback loop. We demonstrate using both ChIP and CHX induction experiments that this feedback loop involves asymmetric direct positive transcriptional activation of *CUC1* by STM and vice versa, such that *CUC1* responds strongly and rapidly to increases in STM activity, whereas the response of *STM* to CUC1 is weaker and slower.

Such a positive-feedback loop appears paradoxical given the distinctly different expression patterns of *STM* and *CUC1*, with STM detected throughout the SAM, except in organ primordia, and CUC1 localised to the meristem-organ boundary zone. With the further finding that STM regulates *miR164c*, a member of the CUC-regulating miR164 family [of which *miR164a/b* are expressed in leaf primordia under control of TCPs and *miR164c* in the SAM in a similar pattern to *STM* (Mallory et al., 2004; Laufs et al., 2004; Baker et al., 2005; Sieber et al., 2007; Koyama et al., 2010)], we used ODE modelling in a spatial context to predict the outcome of the regulatory interactions. Attenuation of the STM-CUC1 feedback loop by STM-induced *miR164c*, coupled with diffusion/movement of STM between cells to create an instructional gradient can explain how observed *STM* and *CUC1* expression patterns can arise from this positive-feedback loop. This patterning is dependent on intercellular movement of STM protein, and movement of KNOX1 proteins through plasmodesmata is well-established and essential for meristem function (Lucas et al., 1995; Jackson, 2002; Kim et al., 2002, 2003; Xu et al., 2011). Importantly, a recent study has demonstrated the crucial role for STM protein movement in establishing the correct expression pattern of CUC1 and CUC2 at the meristem-organ boundary zone (Balkunde et al., 2017) in line with the predictions of the model. Hence, the model provides mechanistic insight into these observations, demonstrating why STM protein movement may be essential for normal SAM patterning and function. The pattern does not require, but is reinforced by, autoregulation of *STM*.

The model can also explain the embryonic patterns of expression. *CUC1* is expressed before *STM* in the embryonic SAM and is required to activate *STM* expression. Initially, their expression is coincident before *CUC1* becomes localised to presumptive meristem-organ boundary zones, in line with model predictions.

Our model describes the regulatory interactions that lead to the establishment of incipient organ boundaries during the early stages of organ primordium formation, and hence does not recapitulate the observed expression patterns during the later stages of primordium development, notably the accumulation of STM in established organ boundaries associated with later-stage organ primordia. This may result from mechanical forces generated between the outgrowing primordium and the meristem (Landrein et al., 2015), symplastic isolation between primordium and meristem (Bayer et al., 2008), or transactivation by CUC1 after boundary establishment. The localisation of STM, a strong activator of *CUC1* expression, to such boundaries could explain why the miR164-resistant CUC1 reporter (pCUC1:mCUC1-GFP) and pCUC1:GUS reporter gene showed strong expression in established organ boundaries in addition to weaker expression throughout the meristem (Sieber et al., 2007), although additional boundary-reinforcing factors are likely also involved (Aida and Tasaka, 2006).

We note that the observed expression of pCUC1:mCUC1-GUS in the central zone, although detectable, is weaker than our model predicts. This might arise due to incomplete resistance of mCUC1 to microRNAs of the miR164 family, as supported by the observed broader expression of pCUC1:GUS throughout the meristem, or might suggest additional transcriptional control mechanisms. A recent study has shown that accumulation of a full-length genomic CUC1-RFP fusion protein in boundaries requires STM mobility and gradient formation, as in non-mobile STM plants CUC1-RFP accumulated throughout the meristem, exactly in line with our model predictions (Balkunde et al., 2017). Therefore, despite the limitations highlighted by potential involvement of additional factors, our model shows that regulatory interactions between a limited set of components (STM, CUC1, miR164c and TCPs) can potentially explain how the different expression patterns of STM and CUC1, and hence the initial delineation between the meristem and boundary zones, can arise.

Positive or double-negative feedback loops are often used to convert graded inputs into switch-like bistable responses (Ferrell, 2002). Positive feedback often involves auto-regulation, and although more complex loops are likely to play key roles in developmental processes, their molecular implementation and dynamics are not well understood. A described example of direct positive feedback between transcription factors is in male sex determination between SOX9 and ER71/ETV2, which are activated by SRY and then participate in an autoregulatory and self-sustaining loop (DiTacchio et al., 2012). The model presented can recapitulate the *STM* and *CUC1* expression domains based on simple interactions between the demonstrated players comprising a positive-feedback loop and coupled negative feedback by a miRNA. An instructional gradient of STM dependent on its diffusion across the SAM gives rise to an output that can be considered as a biphasic switch whose readout is seen in the spatial rather than the temporal domain, generating boundary-specific expression of *CUC1*.

**Fig. 6.**
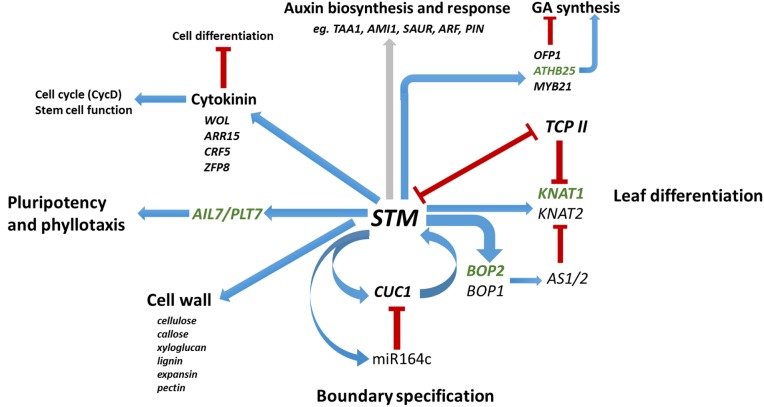
**STM: a nexus in SAM regulation.** Summary of novel *STM* regulatory relationships revealed in this study and previously. Blue arrows indicate positive regulation, red bars indicate negative regulation. Grey arrow indicates more-complex regulation involving up- or downregulation of different genes. Genes in green were identified as direct STM targets in this study.

We conclude that the STM coordinates multiple aspects of SAM function through its GRN of transcription factors, including TCP-mediated control of organ formation and differentiation, *AIL7/PLT7*-mediated regulation of pluripotency and phyllotaxis, and the establishment of meristem-organ boundary zones via *CUC1*, in addition to regulating genes involved in cell wall modification and hormone synthesis and response [as shown in previous studies (Fig. 6)]. This understanding of the range of SAM functions coordinated through the *STM* GRN reinforces its central importance in development and we provide here an understanding of how this is integrated into the wider framework of developmental regulation.

## MATERIALS AND METHODS

### Plant lines and growth conditions

Lines for inducible expression of *STM* (STMoe) and STM-RNAi based on the TGV system have been described previously (Scofield et al., 2013). Controls were TGV empty vector lines. Plants were grown *in vitro* (continuous white light, 22°C) on GM (4.4 g/l MS salts, 1.5% sucrose, 1% agar). Induction was by transfer to medium with 60 µM dexamethasone (DEX) or application of DEX solution (60 µM) to agar surface (short-term experiments). To induce p35S::STM-GR (Brand et al., 2002), 60 µM cycloheximide was added with DEX when applicable. pRPS5A:mCUC1-GR (CUC1-GR; Takeda et al., 2011), pSTM::STM-VENUS (Balkunde et al., 2017), pSTM:GUS (Kirch et al., 2003) and pmiR164c::VENUS (Sieber et al., 2007) have been described. To generate pCUC1:GUS, a 1.7 kb CUC1 promoter fragment was cloned into pBI101. GUS staining was as described by Scofield et al. (2013).

### qRT-PCR analysis

Total RNA was isolated with Tripure (Roche) and cDNA synthesis was performed using the Ambion Retroscript kit. qRT-PCR (Rotorgene 6000; Corbett Research) used Abgene SYBR green mix (Thermo Fisher) and *ACTIN2* as reference. Data was analysed using the ΔΔCT method (Livak and Schmittgen, 2001). All data are relative to induced empty vector or GUS control lines. Averages and standard deviations (error bars) from multiple experiments are shown. Primer sequences are provided in Table S3.

### Microarray analysis

Affymetrix ATH1 DNA microarrays were used for microarray analysis. Raw data were processed using RMA and quantile normalisation (Irizarry et al., 2003) and LIMMA (Smyth, 2005) to identify significantly differentially expressed genes (*P*<0.01). The Benjamini and Hochberg test correction (Benjamini and Hochberg, 1995) was then used to compute adjusted *P*-values with a threshold of <0.01. Timecourse microarray data are available through Array Express/Annotare under accession number E-MTAB-6123. Direct target (cycloheximide) microarray data are available in NASC (The European Arabidopsis Stock Centre; affymetrix.arabidopsis.info) under accession/experiment number 592.

### Chromatin immunoprecipitation (ChIP)

ChIP was performed according to Morohashi et al. (2009), using the anti-glucocorticoid receptor alpha antibody PA1-516 (Thermo Fisher). p35S::STM-GR plants were grown on 60 µM DEX for 9 days and aerial tissue harvested. Data were analysed using fold-enrichment relative to unbound *ACTIN2* control sequence, which showed no enrichment between IP and mock IP samples. Two to five biological replicates were performed per gene. Data were also analysed using a percentage input method yielding similar results. Putative STM-binding sites were identified using the Athamap tool (www.athamap.de) based on the barley HVH21-binding site.

### Meta-analysis

Omnibus *P*-values for the STMoe 8 h, STMoe 24 h, STM-RNAi 72 h and STM-RNAi 9 days samples compared with empty vector controls were calculated using Fisher's inverse chi-squared test as described by Levesque et al. (2006). *P*-values were not separated by the direction of fold-change. Omnibus *P*-values were subsequently corrected by False Discovery Rate correction (Storey, 2002) using the q-value package in R.

### Microarray data preparation for Bayesian network analysis

Using R to connect to the Gene Expression Omnibus via the ArrayExpress Module (Kauffmann et al., 2009), all microarrays annotated for seedling or shoot apex tissue as of 17/3/2013 were obtained (*n*=2373). Arrays annotated as containing root tissue or not at the seedling developmental stage were removed. RMAExpress was used to extract expression values (RMA) and normalise data (quantile normalisation with median polish) from the .cel files. (2003, http://stat-www.berkeley.edu/bolstad/RMAExpress/RMAExpress.html). Expression values for all TFs identified in the meta-analysis were extracted from the dataset and discretised as follows: 2 E_ij_−E_avi_>1; 0 E_avi_−E_ij_>1; 1 otherwise, where E_ij_ is the expression of probe i in condition j and E_avi_ is the expression of probe i over all conditions; i.e. 2 if >2-fold over average expression, 0 if <2-fold than average expression, 1 otherwise.

### Bayesian network structural inference

Using discretised microarray data for the 57 nodes, consensus networks were inferred from the datasets using BANJO version 2 http://www.cs.duke.edu/~amink/software/banjo/. Networks were run for 1 h, with maximum parent count of 5 (constrained for memory considerations), using simulated annealing to search through the solution space, over a maximum of 10,000 restarts, initial simulated annealing temperature of 10,000, a cooling factor of 0.7, reannealing temperature of 800, a maximum of 2500 accepted networks before cooling, a maximum of 10,000 proposed networks before cooling and a minimum of 500 accepted networks before reannealing. Twenty top-scoring networks were generated and a consensus network was produced via influence scores. Networks were visualised using Cytoscape (Shannon et al., 2003).

### GO enrichment analysis

Gene Ontology (GO) enrichment analysis was performed and visualised using BINGO package in Cytoscape using hypergeometric test, *P*<0.05 significance threshold and Benjamini and Hochberg FDR correction. The full Arabidopsis GO annotation sets for biological process, molecular function and cellular component were used in addition to the GOslim annotation files. Input datasets were either DEGs expressed at each time-point in the timecourse, or the meta-analysis dataset of 465 genes.

### Clustering

Hierarchical clustering (average distance UPGMA) of the 465 genes identified in the meta-analysis and *k*-means clustering of the 57 TF-encoding genes was performed using the Epclust Expression Profiler tool (www.ebi.ac.uk).

### Imaging

Fluorescent reporter lines were visualised using a Zeiss 710 meta confocal scanning laser microscope. The 2.5D projection of layer one of the meristem was produced using MorphoGraphX from the confocal image stack (Jones et al., 2017). Cell membranes were stained with FM4-64 and are shown in magenta. Further details can be found in the legend to Fig. 5.

### Mathematical modelling

Modelling was performed and visualised using Python using the ODEs and parameters listed in Appendix S1.

## Supplementary Material

Supplementary information
