# Supplementary material for: Coordination of meristem and boundary functions by transcription factors in the SHOOT MERISTEMLESS regulatory network
Source: Development. 2018 Apr 30;145(9):dev157081. doi: 10.1242/dev.157081 (PMC5992597; doi:10.1242/dev.157081)
Supplement: Supplementary information [file develop-145-157081-s1.pdf]

Appendix S1

Analysis of the SHOOT MERISTEMLESS gene regulatory network reveals coordination of key meristem functions through regulation of transcription factor

Scofield S, Murison A, Jones A, Fozard J, Aida M,
Band L, Bennet M and Murray J

In this text, we provide further details of the development of our mathematical model that seeks to describe key aspects of how STM and CUC1 generate spatial patterning within the shoot apical meristem (SAM).

In the model, we represent a portion of the SAM by a file of N cells; primordia are specified by levels of a Primordium Identity Factor (PRIF) in a subset of these. We aim to determine whether the interaction between STM and CUC1, together with cell-to-cell communication, are sufficient to explain the observed patterns of STM and CUC1 localization. We note that this is deliberately a simple model, omitting effects such as the mechanical regulation of STM transcription [4], which may serve to further refine the spatio-temporal distributions of CUC1 and STM.

1 Model description

The model incorporates the key interactions between proteins and mRNA, as described in the main text and shown in Figure 5 of the main text. In all cells, the transcription of STM mRNA is promoted by CUC1, whereas transcription of CUC1 mRNA is promoted by STM, creating a positive feedback loop. STM promotes the production of the microRNA miRNA164c, which inhibits CUC1 protein production by increasing the degradation rate of CUC1 mRNA. To maintain STM levels in the centre of the meristem, where CUC1 protein levels are thought to be low, we include include STM upregulation of its own transcription and/or basal transcription of STM in the model as optional effects. Both STM and CUC1 mRNA are translated into protein at a constant rate, and the proteins have constant basal degradation rates. However miRNA164c greatly increases the degradation rate of CUC1 mRNA, reducing CUC1 mRNA levels in the centre of the primordium.

Explaining how primordium cells are specified is beyond the scope of the current model; this specification is thought to involve auxin transport and regulation of the PIN auxin efflux carriers and has previously been modelled [3]. As a proxy for this, we introduce a Primordium Identity Factor (PRIF). We prescribe $[\text{PRIF}]_i = 1$ for cells within the primordium, and $[\text{PRIF}]_i = 0$ for the

Development • Supplementary information

remaining cells; this prescribed distribution specifies the primordium's position. Production of TCP is promoted by PRIF. For simplicity we combine the transcription and translation steps of TCP production. TCP further promotes the production of microRNA miRNA164a. As there is no basal production both TCP and miRNA164a are restricted to the primordia. As for miRNA164c, miRNA164a acts through increasing the degradation rate of CUC1 mRNA. TCP is negatively regulated by STM and we suppose that TCP decays at a constant rate.

As the details of transport of STM between neighbouring cells in the meristem are not fully understood (see [5]), we choose a simple model and suppose that the net flux of STM between neighbouring cells is proportional to the concentration difference between them.

1.1 Model equations

We can represent the interactions described above using the a system of $7N$ ordinary differential equations which indicate how the concentrations of each species within each cell (as listed in Table 1) change with time:

$$\frac{\mathrm{d}[mSTM]_i}{\mathrm{d}t} = \frac{c_{STM} + \alpha_{STM}(\beta_1[STM]_i + \beta_3[CUC]_i)}{k_{STM} + \beta_1[STM]_i + \beta_2[TCP]_i + \beta_3[CUC]_i} - \lambda_{STM}[mSTM]_i, \tag{1a}$$

$$\frac{\mathrm{d}[STM]_i}{\mathrm{d}t} = \eta_{STM}[mSTM]_i - \delta_{STM}[STM]_i + P([STM]_{i+1} - 2[STM]_i + [STM]_{i-1}), \tag{1b}$$

$$\frac{\mathrm{d}[mCUC]_i}{\mathrm{d}t} = \frac{\alpha_{CUC}[STM]_i}{k_{CUC} + [STM]_i} - (\lambda_{CUC} + \lambda_{CUCmc}[mc]_i + \lambda_{CUCma}[ma]_i)[mCUC]_i, \tag{1c}$$

$$\frac{\mathrm{d}[CUC]_i}{\mathrm{d}t} = \eta_{CUC}[mCUC]_i - \delta_{CUC}[CUC]_i, \tag{1d}$$

$$\frac{\mathrm{d}[mc]_i}{\mathrm{d}t} = \frac{\alpha_{mc}[STM]_i^{n_c}}{k_{mc}^{n_c} + [STM]_i^{n_c}} - \lambda_{mc}[mc]_i, \tag{1e}$$

$$\frac{\mathrm{d}[ma]_i}{\mathrm{d}t} = \alpha_{ma}[TCP]_i - \lambda_{ma}[ma]_i, \tag{1f}$$

$$\frac{\mathrm{d}[TCP]_i}{\mathrm{d}t} = \frac{\alpha_{TCP}[PRIF]_i}{k_{TCP} + [PRIF]_i + \beta_4[STM]_i} - \delta_{TCP}[TCP]_i, \tag{1g}$$

where $i = 1, \cdots, N$. The model depends on 28 parameters, as listed in Table 2.

We specify the three cells at the left-hand side of the file to be primordium cells, setting levels of the primordium interaction factor to be

$$[\mathrm{PRIF}]_\mathrm{i} = 1 \quad \text{for} \quad i = 1, 2, 3, \qquad [\mathrm{PRIF}]_\mathrm{i} = 0 \quad \text{otherwise.}$$

We assume that the boundaries at either end of the file are impenetrable, which corresponds to the boundary conditions $[STM]_0 = [STM]_1$ and $[STM]_{N+1} = [STM]_N$ (for calculation of the terms in (1b)).

Variable	Description
$[mSTM]_i$	STM mRNA
$[STM]_i$	STM protein
$[mCUC]_i$	CUC mRNA
$[CUC]_i$	CUC1 protein
$[mc]_i$	microRNA, miRNA164c
$[ma]_i$	microRNA, miRNA164a
$[TCP]_i$	TCP protein

Table 1: Model variables for each cell $i = 1, \ldots, N$.

1.2 Parameter values and initial conditions

The model predictions depend on a number of parameters (summarised in Table 2). As we are primarily interested in the steady-state behaviour of this system, and in particular the relative spatial expression patterns, rather than in the absolute concentrations of the network components, all parameter values are dimensionless. We investigated how the choice of parameters affects the predicted distributions of the network components; we found that the proposed network of interactions could qualitatively mimic the biologically observed distributions using the parameter values listed in Table 2. These parameters were selected from a heuristic search of parameter space, selecting parameter values which generated large differences in CUC1 expression levels between the boundary region and the centre of the primordium.

As initial conditions, the STM protein and mRNA concentrations are set equal to 1 for all cells, and the concentrations of all other species set to zero:

$$[STM]_i = [mSTM] = 1, \quad [mCUC]_i = [CUC]_i = [mc]_i = [ma]_i = [TCP]_i = 0 \quad \text{at} \quad t = 0. \quad (2)$$

1.3 Numerical solution

Steady-state solutions were obtained by the numerical integration of equations (1), with initial conditions (2) using standard ODE solvers ([2]), until $T = 10^8$. Python code to reproduce the simulation outputs shown in the paper is attached as a Supplementary File.

1.4 Parameter sensitivity analysis

To assess the sensitivity of simulation outputs to parameter choices, we performed a basic local parametric sensitivity analysis [1]. For each parameter p, we calculated the mean normalized sensitivity $\bar{S}_p$ from

$$\bar{S}_p = \frac{1}{7N} \sum_{i=1}^{N} \sum_{j=1}^{7} \frac{p}{[x_j]_i} \frac{\partial [x_j]_i}{\partial p} \quad (3)$$

where $[x_1]_i = [mSTM]_i$, $[x_2]_i = [STM]_i$, $[x_3]_i = [mCUC]_i$, $[x_4]_i = [CUC]_i$, $[x_5]_i = [mc]_i$, $[x_6]_i = [ma]_i$, $[x_7]_i = [TCP]_i$. Derivatives were calculated through making a small (0.01) relative perturbation in the parameter values.

Development • Supplementary information

Parameter	Description	Value
N	Number of cells	10
c_{STM}	basal STM transcription rate	3 or 0*
λ_{STMm}	STM mRNA degradation rate	1
λ_{CUCm}	CUC1 mRNA degradation rate	1
λ_{mc}	miRNA 164a degradation rate	1
λ_{ma}	miRNA 164c degradation rate	1
λ_{CUCma}	miRNA-164a-enhanced CUC1 mRNA degradation	200
λ_{CUCmc}	miRNA-164c-enhanced CUC1 mRNA degradation	100
k_{STM}	half-saturation constant for STM mRNA transcription	5
k_{CUC}	half-saturation constant for CUC1 mRNA transcription	10
k_{mc}	half-saturation constant for miRNA 164c production	0.8
k_{TCP}	half-saturation constant for TCP production	1
n_c	Hill coefficient for miRNA 164c production	5
η_{STM}	STM translation rate	1
η_{CUC}	CUC1 translation rate	1
β_1	STM autoregulation	0 or 2.5*
β_2	STM inhibition by PRIF	100
β_3	STM regulation by CUC1	0.5
β_4	TCP inhibition by STM	1
α_{ma}	miRNA 164a transcription rate due to TCP	10
α_{mc}	maximum miRNA 164c transcription rate	10
α_{STM}	maximum (non-basal) STM transcription rate	2
α_{CUC}	maximum CUC1 transcription rate	1000
α_{TCP}	maximum TCP production rate	1
δ_{STM}	STM protein degradation rate	1
δ_{CUC}	CUC1 protein degradation rate	1
δ_{TCP}	TCP protein degradation rate	1
P	STM diffusion rate	3 or 0**

Table 2: Model parameters. * Parameter values used for model with STM autoregulation. ** Parameter values used for model without STM transport.

Development • Supplementary information

For simulations with STM autoregulation and no basal STM transcription, as shown in Figure 1, simulation results were particularly sensitive to δ_{STM}, α_{STM}, λ_{STM}, η_{STM}, k_{STM} and β_1; these parameters control the overall level of STM protein. Simulations are also sensitive to k_{mc} and n_c, which regulate the sharp response of miR164c to STM. Without STM autoregulation, but with basal STM transcription, the results of the sensitivity analysis are shown in Figure 2. Simulation outputs are now sensitive to the basal STM transcription rate C_{STM}, but here $\beta_1 = 0$.

Development • Supplementary information

References

[1] DM Hamby. A review of techniques for parameter sensitivity analysis of environmental models. *Environmental monitoring and assessment*, 32(2):135–154, 1994.

[2] A.C. Hindmarsh. ODEPACK, a systematized collection of ODE solvers. In R. S. Stepleman et al., editor, *Scientific Computing*, pages 55–64. North-Holland, Amsterdam, 1983.

[3] Henrik Jönsson, Marcus G. Heisler, Bruce E. Shapiro, Elliot M. Meyerowitz, and Eric Mjolsness. An auxin-driven polarized transport model for phyllotaxis. *Proceedings of the National Academy of Sciences*, 103:1633–1638, 2006.

[4] Benoît Landrein, Annamaria Kiss, Massimiliano Sassi, Aurélie Chauvet, Pradeep Das, Millan Cortizo, Patrick Laufs, Seiji Takeda, Mitsuhiro Aida, Jan Traas, Teva Vernoux, Arezki Boudaoud, and Olivier Hamant. Mechanical stress contributes to the expression of the *STM* homeobox gene in arabidopsis shoot meristems. *eLife*, 4:e07811, 2015.

[5] Xianfeng Morgan Xu, Jing Wang, Zhenyu Xuan, Alexander Goldshmidt, Philippa G. M. Borrill, Nisha Hariharan, Jae Yean Kim, and David Jackson. Chaperonins facilitate knotted1 cell-to-cell trafficking and stem cell function. *Science*, 333:1141–1144, 2011.

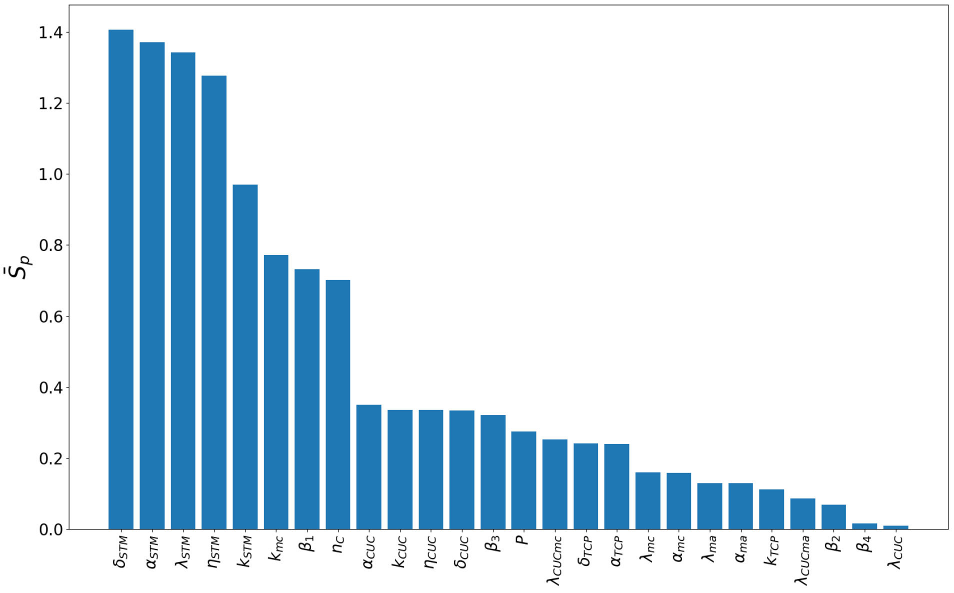

Figure 1: Local sensitivity analysis for steady-state solutions of model with STM autoregulation and no basal STM transcription. Mean normalized sensitivity $\bar{S}_p$ indicates the relative sensitivity of to each of the parameters.

Development • Supplementary information

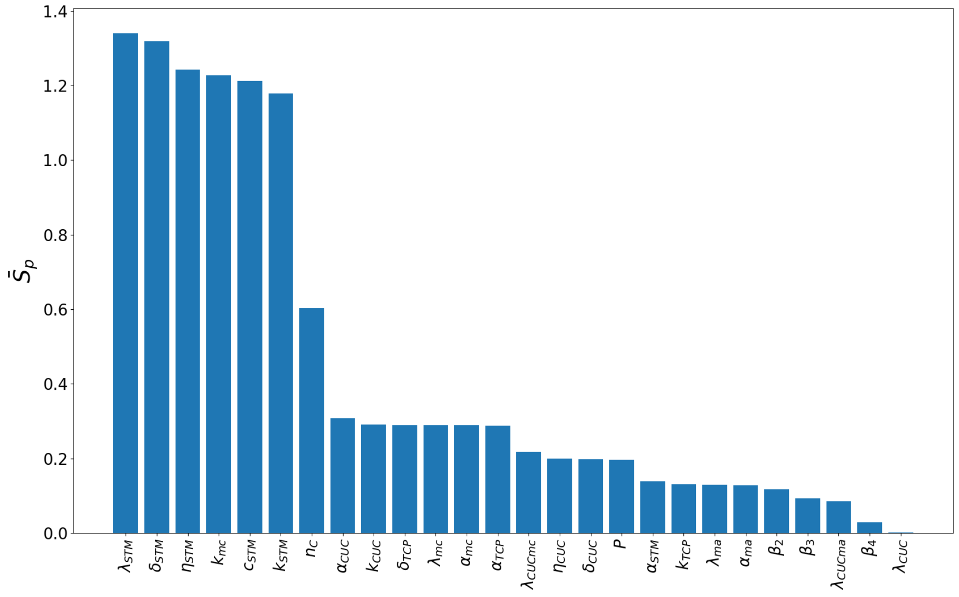

Figure 2: Local sensitivity analysis for steady-state solutions of model with no STM autoregulation and basal STM transcription. Mean normalized sensitivity $\bar{S}_p$ indicates the relative sensitivity of to each of the parameters.

Development • Supplementary information

Table S1. Genes identified using GO analysis.

Genes identified in GO Slim analysis of timecourse data (STMoe 8h, 24h, 72h and 9d) and GO analysis of meta-analysis dataset (465 genes) are shown.

Click here to Download Table S1

Table S2. Differentially expressed genes in STMoe timecourse.

Genes showing significant change in expression (p<0.01) between DEX-treated STMoe and empty vector control lines at 8h, 24h, 72h and 9d timepoints or between DEX-treated STMRNAi lines and empty vector control lines at 72h and 9d are shown. DEGs identified in 3h STM-GR mock versus DEX and cycloheximide versus cycloheximide+DEX and also shown (p<0.01). 3 biological replicates were performed for each timepoint.

Click here to Download Table S2

Development • Supplementary information

Gene:	Forward primer (5'-3'):	Reverse primer (5'-3'):
ACTIN2	acattgtgctcagtggtgga	gtcagggaagcaagaatgga
STM	ggctggaccagaaacagataa	aaagcatggtggaggagatg
CUC1	tcctccgctaaggatgaatg	gagcgggaaggaatgtatga
AIL7	cggaacctttgcaaccgaa	agaagaggacggaagcaact
BOP2	gccaagcatctccctattga	tccaaagctcgtctcatcct
KNAT1/BP	tggccatatccttctgagtc	ccatccatcaccatgaactg
BLH8	agtccgagccatgaacaaac	atttggcttccagttccaaa
OFP1	ggagtcgatggaggagatga	tggggtggtggaagattaag
CHR40	gttattgggccagcaacatt	tgctccatgatcagcttcag
TCP4	ggagcaaccgatacaggaaa	tggagatggattggtgatga
TCP3	cgaggcaatacacagaacca	tctttccaaattccggtgac
MYB21	cacgtcgagccataatgtct	ccaaatatcatccatgcccca
HB25	acggctgagcaaaaggaaag	gatgaagtggtgaaggcgac
pri-miR164c	gcgaacacaaatgaaatcg	agacacgtgttggagtagtag

Table S3. Oligonucleotide primers used for qRT-PCR

Locus ID	Gene Name	Q Value	Description
At1g01070	AT1G01070	0.00069	nodulin MtN21 /EamA-like transporter family protein
At1g01600	CYP86A4	0.00347	CYP86A4; fatty acid (omega-1)-hydroxylase/ oxygen binding; cytochrome p450
At1g01750	ADF11	0.00298	ADF11 (ACTIN DEPOLYMERIZING FACTOR 11); actin binding
At1g02205	CER1	0.00054	CER1 (ECERIFERUM 1); octadecanal decarbonylase
At1g03060	SPI	0.00818	WD/BEACH domain protein SPIRRIG
At1g03240	AT1G03240	0.00098	hypothetical protein
At1g03550	AT1G03550	5.5E-06	secretory carrier-associated membrane protein 2
At1g04050	SUVR1	0.00049	SUVR1 (ARABIDOPSIS HOMOLOG OF SU(VAR)3-9 1); histone-lysine N-methyltransferase/ zinc ion binding
At1g04620	AT1G04620	4.3E-06	7-hydroxymethyl chlorophyll a reductase
At1g05250	AtPRX1/ PRX2	0.00165	peroxidase
At1g05570	CALS1	0.00375	CALS1 (CALLOSE SYNTHASE 1); 1,3-beta-glucan synthase/ transferase, transferring glycosyl groups
At1g05710	AT1G05710	0.00016	basic helix-loop-helix domain-containing protein
At1g06470	AT1G06470	0.00989	probable sugar phosphate/phosphate translocator
At1g06490	CALS7/ ATGSL07	0.00131	CALS7/ ATGSL07 (glucan synthase-like 7); 1,3-beta-glucan synthase/ transferase, transferring glycosyl groups
At1g07640	OBP2	0.00062	OBP2; DNA binding / transcription factor
At1g08980	AMI1	0.00052	AMI1 (AMIDASE 1); amidase/ hydrolase, acting on carbon-nitrogen (but not peptide) bonds / indoleacetamide hydrolase
At1g09530	PIF3	0.00995	PIF3 (PHYTOCHROME INTERACTING FACTOR 3); DNA binding / protein binding / transcription factor/ transcription regulator
At1g10070	ATBCAT-2	0.00019	ATBCAT-2 (ARABIDOPSIS THALIANA BRANCHED-CHAIN AMINO ACID TRANSAMINASE 2); branched-chain-amino-acid transaminase/ catalytic
At1g11080	scpl31	0.00265	scpl31 (serine carboxypeptidase-like 31); serine-type carboxypeptidase
At1g11130	SUB	0.00047	SUB (STRUBBELIG); protein binding / receptor signaling protein serine/threonine kinase
At1g11420	DUF2	0.00079	hypothetical protein
At1g11880	AT1G11880	0.00269	transferases, transferring hexosyl groups
At1g12080	AT1G12080	0.00849	vacuolar calcium-binding protein-like protein
At1g12200	AT1G12200	0.00485	putative flavin monooxygenase.
At1g12320	AT1G12320	0	hypothetical protein
At1g12430	ARK3	0.00367	ARK3 (ARMADILLO REPEAT KINESIN 3); ATP binding / binding / microtubule motor
At1g13400	NUB	0.0018	NUB (NUBBIN); nucleic acid binding / zinc ion binding
At1g13600	AtbZIP58	0.00089	AtbZIP58 (Arabidopsis thaliana basic leucine-zipper 58); DNA binding / transcription factor
At1g15000	scpl50	0.00681	scpl50 (serine carboxypeptidase-like 50); serine-type carboxypeptidase
At1g15250	AT1G15250	0.00036	60S ribosomal protein L37-1
At1g15270	AT1G15270	0.00995	translation machinery associated protein TMA7
At1g15920	AT1G15920	0.00846	polynucleotidyl transferase, ribonuclease H-like superfamily protein
At1g16190	RAD23A	0.00056	DNA repair protein RAD23A
At1g16260	AT1G16260	0.00411	wall-associated receptor kinase-like 8

Development • Supplementary information

At1g16370	OCT6	0.00588	OCT6 (ORGANIC CATION/CARNITINE TRANSPORTER 6); carbohydrate transmembrane transporter/ sugar:hydrogen symporter
At1g16510	AT1G16510	0.00079	SAUR-like auxin-responsive protein
At1g17020	SRG1	0	SRG1 (SENESCENCE-RELATED GENE 1); oxidoreductase
At1g17340	AT1G17340	0.00565	phosphoinositide phosphatase family protein
At1g17700	PRA1.F1	0.00018	PRA1.F1 (PRENYLATED RAB ACCEPTOR 1.F1)
At1g18320	MEE67	0.00415	MEE67 (maternal effect embryo arrest 67); P-P-bond-hydrolysis-driven protein transmembrane transporter/ protein transporter
At1g19950	HVA22H	0.00323	HVA22H (HVA22-LIKE PROTEIN H (ATHVA22H)); ABA response
At1g19960	AT1G19960	0.00082	hypothetical protein
At1g20190	ATEXPA11	1.9E-07	ATEXPA11 (ARABIDOPSIS THALIANA EXPANSIN 11)
At1g21140	AT1G21140	0.00224	vacuolar iron transporter homolog 1
At1g22160	AT1G22160	0.00027	hypothetical protein
At1g22900	AT1G22900	0	disease resistance-responsive, dirigent domain-containing protein
At1g23090	AST91	0.00146	AST91 (SULFATE TRANSPORTER 91); sulfate transmembrane transporter
At1g23800	ALDH2B7	9.6E-06	ALDH2B7; 3-chloroallyl aldehyde dehydrogenase/ aldehyde dehydrogenase (NAD)
At1g24575	AT1G24575	0.00251	hypothetical protein
At1g25260	AT1G25260	0.007	ribosomal protein L10 family protein
At1g25530	AT1G25530	1E-06	transmembrane amino acid transporter family protein
At1g26570	UGD1	0.00813	UGD1 (UDP-GLUCOSE DEHYDROGENASE 1); NAD or NADH binding
At1g27390	TOM20-2	0.00268	TOM20-2 (TRANSLOCASE OUTER MEMBRANE 20-2); P-P-bond-hydrolysis-driven protein transmembrane transporter/ metal ion binding
At1g27980	DPL1	0.00972	DPL1; carboxy-lyase/ catalytic/ pyridoxal phosphate binding
At1g29020	AT1G29020	0.00287	EF-hand, calcium binding motif-containing protein
At1g30300	AT1G30300	0.00156	metallo-beta-lactamase domain-containing protein
At1g30650	WRKY14	0	WRKY14; transcription factor
At1g30820	AT1G30820	0.00169	CTP synthase-like protein
At1g31200	ATPP2-A9	0.00989	ATPP2-A9 (Phloem protein 2-A9); carbohydrate binding
At1g31580	ECS1	0.00185	ECS1; cell wall protein
At1g31690	AT1G31690	0.00041	putative copper amine oxidase
At1g33250	AT1G33250	0.00573	hypothetical protein
At1g33440	AtNPF4.4	0.00053	probable peptide/nitrate transporter NTL1/ PTR family 4.4
At1g34040	AT1G34040	0.00215	Pyridoxal phosphate-dependent transferases superfamily protein /// tryptophan aminotransferase-related protein 4
At1g34510	AT1G34510	0.0073	peroxidase 8
At1g35290	AT1G35290	0	thioesterase family protein
At1g35710	AT1G35710	0.00274	putative leucine-rich repeat receptor-like protein kinase
At1g37170	At1g37170	0.00177	mutator-like transposase
At1g45145	ATTRX5	0.00326	ATTRX5; thioredoxin; oxidoreductase, acting on sulfur group of donors, disulfide as acceptor

At1g47210	CYCA3;2	0.00787	cyclin family protein
At1g48670	AT1G48670	0.00504	auxin-responsive GH3 family protein
At1g49000	AT1G49000	0.00307	hypothetical protein
At1g49470	AT1G49470	0	hypothetical protein
At1g50560	CYP705A25	0.00656	CYP705A25; cytochrome P450; electron carrier/ heme binding / iron ion binding / monooxygenase/ oxygen binding
At1g50630	AT1G50630	8.2E-05	hypothetical protein
At1g51830	AT1G51830	0.00568	putative leucine-rich repeat protein kinase
At1g51960	IQD27	9.3E-05	IQD27 (IQ-domain 27); calmodulin binding
At1g52690	AT1G52690	0	Late embryogenesis abundant protein (LEA) family protein
At1g53120	AT1G53120	0.00565	RNA-binding S4 domain-containing protein
At1g53230	TCP3	0.00365	TCP3; transcription factor
At1g54890	AT1G54890	0.003	Late embryogenesis abundant-related protein
At1g55020	LOX1	0.00686	LOX1; lipoxygenase
At1g55200	AT1G55200	2.9E-05	protein kinase-like protein
At1g55380	EDA11	0.00818	EDA11 (embryo sac development arrest 11); protein binding / zinc ion binding
At1g56120	AT1G56120	0.00461	Leucine-rich repeat transmembrane serine/threonine protein kinase
At1g56710	AT1G56710	8.9E-06	polygalacturonase; pectin lyase-like protein
At1g57680	AT1G57680	0.0006	hypothetical protein
At1g58025	AT1G58025	0.00298	DNA-binding bromodomain-containing protein
At1g58360	AAP1	0.003	AAP1 (AMINO ACID PERMEASE 1); amino acid transmembrane transporter/ neutral amino acid transmembrane transporter
At1g59510	CF9	0.00493	CF9
At1g59820	ALA3	0.00066	ALA3 (Aminophospholipid ATPase3); ATPase, coupled to transmembrane movement of ions,
At1g60060	AT1G60060	0.00568	serine/threonine-protein kinase WNK (With No lysine)-related protein
At1g61130	SCPL32	0.00665	SCPL32 (SERINE CARBOXYPEPTIDASE-LIKE 32); serine-type carboxypeptidase
At1g61790	AT1G61790	0.003	oligosaccharyltransferase complex/magnesium transporter family protein
At1g61810	BGLU45	0	BGLU45 (BETA-GLUCOSIDASE 45); catalytic/ cation binding / hydrolase, hydrolyzing O-glycosyl compounds
At1g62360	STM	0	STM (SHOOT MERISTEMLESS); homeodomain transcription factor
At1g62480	AT1G62480	0.00125	vacuolar calcium-binding protein-like protein
At1g62560	FMO GS-OX3	1.3E-06	FMO GS-OX3 (FLAVIN-MONOOXYGENASE GLUCOSINOLATE S-OXYGENASE 3);
At1g64170	ATCHX16	0.0071	ATCHX16 (CATION/H+ EXCHANGER 16); monovalent cation:proton antiporter/ sodium:hydrogen antiporter
At1g64370	AT1G64370	0	hypothetical protein
At1g64410	At1g64410	0	hypothetical protein
At1g65450	GLC	0.00065	dual transcription unit and alternative splicing protein GLAUCE
At1g65480	FT	0.00333	FT (FLOWERING LOCUS T); phosphatidylethanolamine binding / protein binding
At1g66280	BGLU22	0.00198	BGLU22; catalytic/ cation binding / hydrolase, hydrolyzing O-glycosyl compounds

At1g66730	LIG6	0	DNA ligase 6
At1g67570	AT1G67570	0.00148	hypothetical protein
At1g67860	AT1G67860	0	hypothetical protein
At1g68710	AT1G68710	0.00693	putative phospholipid-transporting ATPase 9
At1g68850	AT1G68850	0.0052	peroxidase 11
At1g69080	AT1G69080	6.1E-05	adenine nucleotide alpha hydrolases-like protein
At1g69526	AT1G69526	0.00935	S-adenosyl-L-methionine-dependent methyltransferases superfamily protein
At1g70210	CYCD1;1	0	CYCD1;1 (CYCLIN D1;1); cyclin-dependent protein kinase regulator
At1g70510	KNAT2	0.00104	KNAT2 (KNOTTED-LIKE FROM ARABIDOPSIS THALIANA 2); transcription factor
At1g70560	TAA1	9.5E-06	TAA1 (TRYPTOPHAN AMINOTRANSFERASE OF ARABIDOPSIS 1);
At1g70670	CLO4	0.00301	probable peroxygenase 4; Caleosin-related family protein
At1g70890	MLP43	0.00476	MLP43 (MLP-LIKE PROTEIN 43)
At1g71820	SEC6	0.00051	SEC6
At1g72930	TIR	0.00814	TIR (TOLL/INTERLEUKIN-1 RECEPTOR-LIKE); transmembrane receptor
At1g73040	AT1G73040	0.00228	Mannose-binding lectin superfamily protein
At1g73177	BNS	0.00378	BNS (BONSAI); anaphase-promoting complex 13
At1g73620	AT1G73620	1.2E-05	pathogenesis-related thaumatin-like protein
At1g74010	AT1G74010	0.00422	strictosidine synthase-like protein
At1g74500	TMO7	0.00041	target of monopteros 7; transcription factor bHLH135
At1g74890	ARR15	0.00601	ARR15 (RESPONSE REGULATOR 15); transcription regulator/ two-component response regulator
At1g75300	AT1G75300	0.00656	isoflavone reductase-like protein
At1g75390	AtbZIP44	0.00041	AtbZIP44 (Arabidopsis thaliana basic leucine-zipper 44); DNA binding / protein heterodimerization/ transcription factor
At1g75780	TUB1	0.00074	TUB1; GTP binding / GTPase/ structural molecule
At1g76130	AMY2	0.00637	AMY2 (ALPHA-AMYLASE-LIKE 2); alpha-amylase/ calcium ion binding / catalytic/ cation binding
At1g76420	CUC3	0	CUC3 (CUP SHAPED COTYLEDON3); transcription factor
At1g76930	ATEXT4	0.00083	ATEXT4 (EXTENSIN 4); structural constituent of cell wall
At1g78080	RAP2.4	0.00818	RAP2.4 (related to AP2 4); DNA binding / transcription factor
At1g78450	AT1G78450	0.00476	SOUL/heme-binding protein-like protein
At1g78970	LUP1	0.00538	LUP1 (LUPEOL SYNTHASE 1); beta-amyrin synthase/ lupeol synthase
At1g79000	HAC1	0.00952	HAC1 (HISTONE ACETYLTRANSFERASE OF THE CBP FAMILY 1); H3/H4 histone acetyltransferase/ transcription cofactor
At1g79970	AT1G79970	0	hypothetical protein
At1g80050	APT2	0.00036	APT2 (ADENINE PHOSPHORIBOSYL TRANSFERASE 2); adenine phosphoribosyltransferase/ phosphate transmembrane transporter
At1g80450	AT1G80450	0.00959	VQ motif-containing protein
At1g80550	AT1G80550	0.00098	pentatricopeptide repeat-containing protein
At1g80830	NRAMP1	5.4E-06	NRAMP1 (NATURAL RESISTANCE-ASSOCIATED MACROPHAGE PROTEIN 1); inorganic anion transmembrane transporterr

At2g01210	AT2G01210	0.00439	leucine-rich repeat transmembrane protein kinase-like protein
At2g01740	AT2G01740	0.00818	pentatricopeptide repeat-containing protein
At2g01830	WOL	8.7E-05	WOL (WOODEN LEG); cytokinin receptor/ osmosensor/ phosphoprotein phosphatase/ protein histidine kinase
At2g01990	AT2G01990	0	hypothetical protein
At2g02130	LCR68	0	LCR68 (LOW-MOLECULAR-WEIGHT CYSTEINE-RICH 68); peptidase inhibitor
At2g03200	AT2G03200	0.00078	aspartyl protease family protein
At2g03890	PI4K GAMMA 7	0.00011	phosphoinositide 4-kinase gamma 7
At2g04270	RNEE/G	0.0031	RNEE/G (RNASE E/G-LIKE); endoribonuclease
At2g07680	ATMRP11	0.00698	ATMRP11; ATPase, coupled to transmembrane movement of substances
At2g14460	AT2G14460	0.00065	hypothetical protein
At2g14560	LURP1	0.00099	LURP1 (LATE UPREGULATED IN RESPONSE TO HYALOPERONOSPORA PARASITICA)
At2g16650	PRORP2	0.00287	antiporter/ drug transporter /// proteinaceous RNase P 2
At2g18260	SYP112	0.00268	SYP112 (SYNTAXIN OF PLANTS 112); SNAP receptor
At2g18480	AT2G18480	0.00907	putative polyol transporter 3
At2g19930	AT2G19930	0	probable RNA-dependent RNA polymerase 5
At2g20340	AT2G20340	0.00701	tyrosine decarboxylase 1
At2g21140	ATPRP2	0.00735	ATPRP2 (PROLINE-RICH PROTEIN 2)
At2g21620	RD2	0	RD2; response to dessication 2
At2g21650	MEE3	0.00041	MEE3 (MATERNAL EFFECT EMBRYO ARREST 3); DNA binding / transcription factor
At2g22190	AT2G22190	0	probable trehalose-phosphate phosphatase E
At2g23620	MES1	3E-05	MES1 (METHYL ESTERASE 1); hydrolase,
At2g23930	SNRNP-G	0.00398	SNRNP-G (PROBABLE SMALL NUCLEAR RIBONUCLEOPROTEIN G)
At2g25210	AT2G25210	0.00901	60S ribosomal protein L39-1 /// 60S ribosomal protein L39-1
At2g25520	AT2G25520	0.00047	putative sugar phosphate/phosphate translocator
At2g26580	YAB5	0.00041	YAB5 (YABBY5); transcription factor
At2g27990	BLH8/ PNF	0	BLH8 (BEL1-LIKE HOMEODOMAIN 8); POUNDFOOLISH (PNF); DNA binding / transcription factor
At2g28230	AT2G28230	0.00848	hypothetical protein /// mediator of RNA polymerase II transcription subunit 20a
At2g28510	AT2G28510	0.00088	Dof zinc finger protein DOF2.1
At2g29750	UGT71C1	0.00021	UGT71C1 (UDP-GLUCOSYL TRANSFERASE 71C1); UDP-glycosyltransferase/ quercetin 3'-O-glucosyltransferase
At2g30870	GSTF10	0.00259	GSTF10 (HALIANA GLUTATHIONE S-TRANSFERASE PHI 10); copper ion binding / glutathione binding / glutathione transferase
At2g30890	AT2G30890	0	Cytochrome b561/ferric reductase transmembrane protein family
At2g31160	LSH3	0	LSH3 (LIGHT SENSITIVE HYPOCOTYLS 3)
At2g31340	emb1381	0.00275	emb1381 (embryo defective 1381)
At2g32690	GRP23	0.00784	GRP23 (GLYCINE-RICH PROTEIN 23)
At2g32970	AT2G32970	0.00059	hypothetical protein

At2g33850	AT2G33850	0.00039	hypothetical protein
At2g34830	WRKY35	0.00074	WRKY35 (WRKY DNA-binding protein 35); transcription factor
At2g35020	GlcNA.1UT2	0.00098	putative UDP-N-acetylglucosamine pyrophosphorylase
At2g35060	KUP11	0.00499	KUP11; potassium ion transmembrane transporter
At2g35750	AT2G35750	0.00013	hypothetical protein
At2g35960	NHL12	0.00051	NHL12; putative harpin-induced protein
At2g36710	AT2G36710	0.00534	putative pectinesterase 15
At2g36870	XTH32	0.00125	probable xyloglucan endotransglucosylase/hydrolase protein 32
At2g37380	AT2G37380	4.6E-05	membrane-associated kinase regulator
At2g37630	AS1	0.0002	AS1 (ASYMMETRIC LEAVES 1); DNA binding / protein homodimerization/ transcription factor
At2g37640	EXP3	0.0017	EXPANSIN3
At2g38080	IRX12	0.0047	IRX12 (IRREGULAR XYLEM 12); laccase
At2g38160	AT2G38160	0.00041	hypothetical protein
At2g38750	ANNAT4	0	ANNAT4 (ANNEXIN ARABIDOPSIS 4); calcium ion binding / calcium-dependent phospholipid binding
At2g38780	AT2G38780	0	hypothetical protein
At2g39190	ATATH8	0.00242	ATATH8; transporter
At2g39230	LOJ	0.00485	pentatricopeptide repeat protein LOJ
At2g39350	AT2G39350	0.00099	ABC transporter G family member 1
At2g39705	RTFL8	0.00726	RTFL8 (ROTUNDIFOLIA LIKE 8)
At2g39980	AT2G39980	0	HXXXD-type acyl-transferase-like protein
At2g41090	AT2G41090	0.00117	calmodulin-like protein 10
At2g41370	BOP2	0	BOP2 (BLADE ON PETIOLE2); protein binding
At2g41940	ZFP8	5E-05	ZFP8 (ZINC FINGER PROTEIN 8); nucleic acid binding / transcription factor/ zinc ion binding
At2g41990	AT2G41990	0.00359	hypothetical protein
At2g42800	AtRLP29	0	AtRLP29 (Receptor Like Protein 29); protein binding
At2g43660	AT2G43660	0	carbohydrate-binding X8 domain-containing protein
At2g44500	AT2G44500	0.0099	O-fucosyltransferase family protein
At2g45810	AT2G45810	2.4E-05	DEAD-box ATP-dependent RNA helicase 6
At2g46310	CRF5	0.00926	CRF5 (CYTOKININ RESPONSE FACTOR 5); AP2 domain DNA binding / transcription factor
At2g46590	DAG2	0.00032	DAG2 (DOF AFFECTING GERMINATION 2); DNA binding / transcription factor
At2g46640	AT2G46640	0	hypothetical protein
At2g47160	BOR1	0.00034	BOR1 (REQUIRES HIGH BORON 1); anion exchanger/ boron transporter
At2g47550	AT2G47550	0.00047	probable pectinesterase/pectinesterase inhibitor 20
At3g01350	AT3G01350	0	putative peptide/nitrate transporter
At3g01750	AT3G01750	0.00139	ankyrin repeat-containing protein

Development • Supplementary information

At3g01960	AT3G01960	0.00101	hypothetical protein
At3g02500	AT3G02500	0	hypothetical protein
At3g03040	AT3G03040	0.00035	F-box protein
At3g03370	AT3G03370	0.00643	hypothetical protein
At3g05730	AT3G05730	0	defensin-like protein 205
At3g05800	AIF1	0.00656	activation-tagged BRI1 suppressor 1-interacting factor 1
At3g05890	RCI2B/ LTI6B	0.00091	RCI2B (RARE-COLD-INDUCIBLE 2B); LTI6B
At3g08840	AT3G08840	0.00224	D-alanine--D-alanine ligase family protein
At3g10590	AT3G10590	0.0005	SANT DNA-binding domain-containing protein
At3g11120	AT3G11120	0.00538	60S ribosomal protein L41
At3g11700	FLA18	0.00034	FLA18 (FASCICLIN-LIKE ARABINOGALACTAN PROTEIN 18 PRECURSOR)
At3g11930	AT3G11930	0	universal stress protein-like protein; Adenine nucleotide alpha hydrolases-like superfamily protein;
At3g13630	AT3G13630	0.00265	hypothetical protein
At3g13845	AT3G13845	0.00629	hypothetical protein
At3g14340	AT3G14340	0.00224	hypothetical protein
At3g14560	AT3G14560	6.6E-06	hypothetical protein
At3g15030	TCP4	9.3E-05	TCP4 (TCP family transcription factor 4); transcription factor
At3g15170	CUC1	0	CUC1 (CUP-SHAPED COTYLEDON1); transcription factor
At3g15720	AT3G15720	0	putative polygalacturonase
At3g15820	ROD1	0.00142	phosphatidic acid phosphatase-related protein /// phosphatidylcholine:diacylglycerol cholinephosphotransferase
At3g16180	AT3G16180	0	probable peptide/nitrate transporter
At3g16390	NSP1	0.006	NSP1 (NITRILE SPECIFIER PROTEIN 1)
At3g16660	AT3G16660	0	pollen Ole e 1 allergen and extensin family protein
At3g16670	AT3G16670	0	pollen Ole e 1 allergen and extensin family protein
At3g16800	AT3G16800	0.0051	putative protein phosphatase 2C
At3g16980	NRPB9A	0.00028	NRPB9A; RNA Polymerase II subunit 14.5 kD; DNA binding / DNA-directed RNA polymerase/ nucleic acid binding / zinc ion binding
At3g18000	XPL1	0.00265	XPL1 (XIPOTL 1); methyltransferase/ phosphoethanolamine N-methyltransferase
At3g18610	ATRANGAP1	0.00686	ATRANGAP1 (RAN GTPASE-ACTIVATING PROTEIN 1); nucleic acid binding / nucleotide binding
At3g19720	ARC5	0.00156	ARC5 (ACCUMULATION AND REPLICATION OF CHLOROPLAST 5); GTP binding / GTPase
At3g20630	UBP14	0.0093	UBP14 (UBIQUITIN-SPECIFIC PROTEASE 14); ubiquitin-specific protease
At3g21950	AT3G21950	0.00298	methyltransferase
At3g22231	PCC1	0.00114	PCC1 (PATHOGEN AND CIRCADIAN CONTROLLED 1)
At3g22425	IGPD	0.00686	IGPD; imidazoleglycerol-phosphate dehydratase
At3g22570	AT3G22570	0.00024	protease inhibitor/seed storage/lipid transfer protein family protein
At3g22670	AT3G22670	0.0092	pentatricopeptide repeat-containing protein

Development • Supplementary information

At3g22740	HMT3	6E-05	HMT3; homocysteine S-methyltransferase
At3g23290	LSH4	0	LSH4 (LIGHT SENSITIVE HYPOCOTYLS 4)
At3g23730	XTH16	0.00027	xyloglucan endotransglucosylase/hydrolase protein 16
At3g24340	chr40	0	chr40 (chromatin remodeling 40); ATP binding / DNA binding / helicase/ nucleic acid binding
At3g24450	AT3G24450	0.0081	heavy metal transport/detoxification domain-containing protein
At3g24490	AT3G24490	0.00298	alcohol dehydrogenase transcription factor myb/SANT-like protein
At3g24660	TMKL1	0.00324	TMKL1 (transmembrane kinase-like 1); ATP binding / kinase/ protein serine/threonine kinase
At3g25540	LAG1	0.00158	LAG1
At3g25940	AT3G25940	0.00696	TFIIB zinc-binding protein
At3g27650	LBD25	0	LBD25 (LOB DOMAIN-CONTAINING PROTEIN 25)
At3g27660	OLEO4	0.00117	OLEO4 (OLEOSIN 4)
At3g27810	ATMYB21	0	ATMYB21 (ARABIDOPSIS THALIANA MYB DOMAIN PROTEIN 21); DNA binding / transcription factor
At3g28007	SWEET4	0.00021	bidirectional sugar transporter SWEET4
At3g42725	AT3G42725	0.00704	putative membrane lipoprotein
At3g43320	At3g43320	0.00656	putative protein retrotransposon finger protein
At3g43800	ATGSTU27	0.00037	ATGSTU27 (GLUTATHIONE S-TRANSFERASE TAU 27); glutathione transferase
At3g43810	CAM7	0	CAM7 (CALMODULIN 7); calcium ion binding
At3g44820	AT3G44820	0.00735	phototropic-responsive NPH3 family protein
At3g45010	scpl48	0.00504	scpl48 (serine carboxypeptidase-like 48); serine-type carboxypeptidase
At3g45450	At3g45450	0.00099	clpC-like protein AtClpC ; ATP binding / ATPase/ nuclease/ nucleoside-triphosphatase
At3g46280	AT3G46280	0.00258	protein kinase-like protein
At3g46490	AT3G46490	0	iron ion binding / oxidoreductase/ oxidoreductase protein
At3g48550	AT3G48550	0.00117	putative zinc-finger protein
At3g48570	AT3G48570	0.00471	protein transport protein sec61 subunit gamma-3
At3g48740	AT3G48740	0	bidirectional sugar transporter SWEET11
At3g49430	SRp34a	0.00022	SRp34a (Ser/Arg-rich protein 34a); RNA binding / nucleic acid binding / nucleotide binding
At3g49810	AT3G49810	2E-05	U-box domain-containing protein 30
At3g50440	MES10	0	MES10 (METHYL ESTERASE 10); hydrolase/ hydrolase, acting on ester bonds / methyl jasmonate esterase
At3g50700	AtIDD2	0.00036	AtIDD2 (Arabidopsis thaliana Indeterminate(ID)-Domain 2); nucleic acid binding / transcription factor/ zinc ion binding
At3g52490	AT3G52490	9.3E-05	heat shock protein-like protein
At3g54000	AT3G54000	0.00415	hypothetical protein
At3g54500	AT3G54500	0.00146	hypothetical protein
At3g54590	ATHRGP1	0.00429	ATHRGP1 (HYDROXYPROLINE-RICH GLYCOPROTEIN); structural constituent of cell wall
At3g54620	BZIP25	0.00466	BZIP25 (BASIC LEUCINE ZIPPER 25); protein heterodimerization/ transcription factor
At3g54820	PIP2;5	2.4E-06	PIP2;5 (PLASMA MEMBRANE INTRINSIC PROTEIN 2;5); water channel

Development • Supplementary information

At3g55710	AT3G55710	0.00153	UDP-glycosyltransferase 76F2
At3g56710	SIB1	8.3E-06	SIB1 (SIGMA FACTOR BINDING PROTEIN 1); binding / protein binding
At3g56810	AT3G56810	0	hypothetical protein
At3g57130	BOP1	0.00013	BOP1 (BLADE ON PETIOLE 1); protein binding
At3g57490	AT3G57490	0.00396	40S ribosomal protein S2-4
At3g57710	AT3G57710	0.00913	protein kinase family protein
At3g57750	AT3G57750	0.00604	putative protein kinase
At3g58510	AT3G58510	0.00041	DEAD-box ATP-dependent RNA helicase 11
At3g58540	AT3G58540	0.00113	hypothetical protein
At3g58640	AT3G58640	0.0084	mitogen activated protein kinase kinase kinase-like protein
At3g58770	AT3G58770	0	hypothetical protein
At3g59010	PME61	0.00169	pectinesterase 35
At3g59270	AT3G59270	0	FBD domain-containing protein
At3g59690	IQD13	0.00104	IQD13 (IQ-domain 13); calmodulin binding
At3g60160	ATMRP9	0.00251	ATMRP9; ATPase, coupled to transmembrane movement of substances
At3g60800	AT3G60800	0.00256	DHHC-type zinc finger family protein
At3g61250	AtMYB17	0.0002	AtMYB17 (myb domain protein 17); DNA binding / transcription factor
At3g61640	AGP20	0.0071	Arabinogalactan protein 20 (AGP20)
At3g62680	PRP3	0.00912	PRP3 (PROLINE-RICH PROTEIN 3); structural constituent of cell wall
At3g62740	BGLU7	0	BGLU7 (BETA GLUCOSIDASE 7); catalytic/ cation binding / hydrolase, hydrolyzing O-glycosyl compounds
At3g63300	FKD1	0.00681	protein forked1
At4g00400	GPAT8	0.00293	GPAT8 (glycerol-3-phosphate acyltransferase 8); acyltransferase/ glycerol-3-phosphate O-acyltransferase
At4g01390	AT4G01390	0	TRAF-like family protein
At4g02130	GATL6	0.00117	GATL6; polygalacturonate 4-alpha-galacturonosyltransferase/ transferase, transferring glycosyl groups / transferase, transferring hexosyl groups
At4g02270	RHS13	1.9E-05	protein root hair specific 13
At4g07950	AT4G07950	9.3E-06	DNA-directed RNA polymerase, subunit M
At4g08150	KNAT1	0.00025	KNAT1 (KNOTTED-LIKE FROM ARABIDOPSIS THALIANA); BP; transcription factor
At4g08290	AT4G08290	0	nodulin MtN21 /EamA-like transporter family protein
At4g08300	AT4G08300	0	nodulin MtN21 /EamA-like transporter family protein
At4g08960	AT4G08960	0.0051	phosphotyrosyl phosphatase activator (PTPA) family protein
At4g09730	RH39	0.0067	DEAD-box ATP-dependent RNA helicase 39
At4g10380	NIP5;1	0.00049	NIP5;1; arsenite transmembrane transporter/ boron transporter/ water channel
At4g11110	SPA2	0.0099	SPA2 (SPA1-RELATED 2); protein binding / signal transducer / photomorphogenesis repressor
At4g14040	SBP1	0.00839	SBP1 (selenium-binding protein 1); selenium binding
At4g14400	ACD6	0.00234	ACD6 (ACCELERATED CELL DEATH 6); protein binding

At4g15140	AT4G15140	0.00864	hypothetical protein
At4g15800	RALFL33	0	RALFL33 (ralf-like 33); signal transducer
At4g16180	AT4G16180	0.00882	hypothetical protein
At4g16447	AT4G16447	0.00655	hypothetical protein
At4g16500	AT4G16500	0	cysteine proteinase inhibitor 4
At4g17030	ATEXLB1	2.3E-05	ATEXLB1 (ARABIDOPSIS THALIANA EXPANSIN-LIKE B1)
At4g17260	AT4G17260	0.00293	L-lactate dehydrogenase
At4g18210	ATPUP10	0.00041	ATPUP10; purine transmembrane transporter
At4g18890	BEH3	0.00117	BES1/BZR1-like protein 3
At4g19790	At4g19790	0.0084	putative LTR retrotransposon; copia-like retrotransposon family
At4g21680	NRT1.8	0.00727	nitrate transporter 1.8
At4g22010	SKS4	0.0091	sks4 (SKU5 Similar 4); copper ion binding / oxidoreductase
At4g22160	AT4G22160	0.00158	hypothetical protein
At4g22300	SOBER1	0.00104	SOBER1 (SUPPRESSOR OF AVRBST-ELICITED RESISTANCE 1); carboxylesterase
At4g22790	AT4G22790	5.5E-05	mate efflux domain-containing protein
At4g23660	AtPPT1	0.00735	AtPPT1 (Arabidopsis thaliana polyprenyltransferase 1); 4-hydroxybenzoate nonaprenyltransferase
At4g25090	AT4G25090	0.00366	riboflavin synthase-like superfamily protein
At4g25760	ATGDU2	0.00125	AtGDU2 (Arabidopsis thaliana GLUTAMINE DUMPER 2)
At4g25910	NFU3	0.00789	NFU3; structural molecule; nitrogen fixation like protein
At4g26130	AT4G26130	0.00098	hypothetical protein
At4g26140	BGAL12	0.00239	BGAL12 (beta-galactosidase 12); beta-galactosidase/ catalytic/ cation binding
At4g26230	AT4G26230	0.00972	60S ribosomal protein L31-2
At4g26320	AGP13	0.0008	AGP13 (arabinogalactan protein 13)
At4g26510	UKL4	0.00036	uridine kinase-like 4
At4g27350	AT4G27350	0.00422	hypothetical protein
At4g27720	AT4G27720	0.00789	major facilitator protein
At4g28400	AT4G28400	0.00179	putative protein phosphatase 2C 58
At4g28650	AT4G28650	0.00246	putative leucine-rich repeat transmembrane protein kinase
At4g28850	XTH26	0.00847	xyloglucan endotransglucosylase/hydrolase 26
At4g29690	AT4G29690	0.00054	alkaline-phosphatase-like protein
At4g31570	AT4G31570	0.0042	hypothetical protein
At4g31805	AT4G31805	0.00062	POLAR protein; asymmetric cell division
At4g31985	AT4G31985	0.00032	60S ribosomal protein L39-1
At4g32070	AT4G32070	0.00026	octicosapeptide/Phox/Bem1p and tetratricopeptide repeat domain-containing protein
At4g32290	AT4G32290	0.00422	core-2/I-branching beta-1,6-N-acetylglucosaminyltransferase family protein

At4g32390	AT4G32390	0.00668	nucleotide-sugar transporter family protein
At4g34400	AT4G34400	0.00228	AP2/B3-like transcriptional factor family protein
At4g34480	AT4G34480	0	glucan endo-1,3-beta-glucosidase 7
At4g35040	bZIP19	0.0084	transcription factor bZIP19
At4g36830	HOS3-1	0.00863	GNS1/SUR4 membrane protein
At4g37150	MES9	0	MES9 (METHYL ESTERASE 9); hydrolase, acting on ester bonds
At4g37250	AT4G37250	0.00168	putative LRR receptor-like serine/threonine-protein kinase
At4g37430	CYP91A2	4.4E-05	CYP91A2 (CYTOCHROME P450 MONOOXYGENASE 91A2); electron carrier/ heme binding / iron ion binding / monooxygenase/ oxygen binding
At4g37530	AT4G37530	1.9E-05	peroxidase family protein
At4g37650	SHR	0.00072	SHR (SHORT ROOT); protein binding / sequence-specific DNA binding / transcription factor
At4g38010	AT4G38010	0.00925	pentatricopeptide repeat-containing protein
At4g38530	ATPLC1	0	ATPLC1 (ARABIDOPSIS THALIANA PHOSPHOLIPASE C 1); phospholipase C
At4g38770	PRP4	0.00251	PRP4 (PROLINE-RICH PROTEIN 4); cell wall protein
At4g39230	ATPCBER1	0.00825	NAD(P)H oxidoreductase, isoflavone reductase
At4g39420	AT4G39420	0.00166	hypothetical protein
At4g39770	TPPH	0.00109	trehalose-phosphate phosphatase H
At5g01840	OFP1	0	OFP1 (OVATE FAMILY PROTEIN 1); protein binding / transcription repressor
At5g04230	PAL3	0	PAL3 (PHENYL ALANINE AMMONIA-LYASE 3); phenylalanine ammonia-lyase
At5g04950	NAS1	2.3E-05	NAS1 (NICOTIANAMINE SYNTHASE 1); nicotianamine synthase
At5g04960	AT5G04960	0.00012	putative pectinesterase/pectinesterase inhibitor 46
At5g05260	CYP79A2	0.00709	CYP79A2 (CYTOCHROME P450 79A2); oxidoreductase
At5g05880	AT5G05880	0.00665	UDP-glycosyltransferase 76C4
At5g06320	NHL3	0.00582	NHL3; harpin-induced protein-like
At5g06610	AT5G06610	0.0071	hypothetical protein
At5g07690	ATMYB29	0.00704	ATMYB29 (ARABIDOPSIS THALIANA MYB DOMAIN PROTEIN 29); DNA binding / transcription factor
At5g07740	At5g07740	0.00426	hypothetical protein
At5g07830	PIP1B	0.00596	PIP1B (NAMED PLASMA MEMBRANE INTRINSIC PROTEIN 1B); water channel
At5g08040	TOM5	0.00098	TOM5 (MITOCHONDRIAL IMPORT RECEPTOR SUBUNIT TOM5 HOMOLOG)
At5g09440	EXL4	0.00668	EXL4 (EXORDIUM LIKE 4)
At5g09510	AT5G09510	0.00686	40S ribosomal protein S15-4
At5g10020	SIRK1	0.00114	SUCROSE-INDUCED RECEPTOR KINASE 1
At5g10100	AT5G10100	1.4E-05	probable trehalose-phosphate phosphatase I
At5g10140	FLC	0.00668	FLC (FLOWERING LOCUS C); specific transcriptional repressor/ transcription factor
At5g10170	MIPS3	0.00779	MIPS3 (MYO-INOSITOL-1-PHOSTPATE SYNTHASE 3); binding / catalytic/ inositol-3-phosphate synthase
At5g10760	AED1	0.0012	aspartyl protease family protein

At5g11320	YUC4	0.00036	YUC4 (YUCCA4); flavinoid monooxygenase/ oxidoreductase
At5g11410	AT5G11410	0.0073	protein kinase family protein
At5g12170	CLT3	0.0011	CRT (chloroquine-resistance transporter)-like transporter 3
At5g13030	AT5G13030	0.00072	hypothetical protein
At5g13580	AT5G13580	0.00559	ABC transporter G family member 6
At5g14090	AT5G14090	0.00907	hypothetical protein
At5g14940	AT5G14940	5.7E-05	probable peptide/nitrate transporter
At5g15580	LNG1	0.00014	LNG1 (LONGIFOLIA1)
At5g15840	CO	0.00153	CO (CONSTANS); transcription factor/ transcription regulator/ zinc ion binding
At5g16080	AtCXE17	0	AtCXE17 (Arabidopsis thaliana carboxyesterase 17); hydrolase
At5g16190	CSLA11	0.00447	ATCSLA11; cellulose synthase/ transferase, transferring glycosyl groups
At5g16530	PIN5	0.00251	PIN5 (PIN-FORMED 5); auxin:hydrogen symporter/ transporter
At5g16600	MYB43	0.00111	MYB43 (myb domain protein 43); DNA binding / transcription factor
At5g16630	RAD4	0.00818	RAD4; damaged DNA binding
At5g16910	CSLD2	0.00846	CSLD2 (CELLULOSE-SYNTHASE LIKE D2); cellulose synthase/ transferase, transferring glycosyl groups
At5g18780	AT5G18780	0.0099	F-box domain-containing protein
At5g19260	AT5G19260	0.00039	hypothetical protein
At5g19520	MSL9	0.00062	MSL9 (MECHANOSENSITIVE CHANNEL OF SMALL CONDUCTANCE-LIKE 9); mechanically-gated ion channel
At5g20470	XIK	0.0018	XIK; motor/ protein binding
At5g20960	AAO1	0.0008	AAO1 (ARABIDOPSIS ALDEHYDE OXIDASE 1); aldehyde oxidase/ indole-3-acetaldehyde oxidase
At5g22390	AT5G22390	5E-05	hypothetical protein
At5g22650	HD2B	0.00028	HD2B (HISTONE DEACETYLASE 2B); histone deacetylase
At5g22860	AT5G22860	1.8E-06	Serine carboxypeptidase S28 family protein
At5g23020	IMS2	3.4E-05	IMS2 (2-ISOPROPYLMALATE SYNTHASE 2); 2-isopropylmalate synthase/ methylthioalkylmalate synthase
At5g23810	AAP7	0	AAP7; amino acid transmembrane transporter
At5g23940	EMB3009	0.00062	EMB3009 (embryo defective 3009); transferase/ transferase, transferring acyl groups other than amino-acyl groups
At5g24030	SLAH3	0.00075	SLAH3 (SLAC1 HOMOLOGUE 3); transporter
At5g24580	AT5G24580	0.00013	heavy metal transport/detoxification domain-containing protein
At5g25490	AT5G25490	0	Ran BP2/NZF zinc finger-like superfamily protein
At5g25640	AT5G25640	0.00277	Rhomboid-related intramembrane serine protease family protein
At5g26280	AT5G26280	0.00847	TRAF-like family protein
At5g27920	AT5G27920	0.00296	F-box protein
At5g27930	AT5G27930	0.00228	putative protein phosphatase 2C 73
At5g28490	LSH1	0.00955	LSH1 (LIGHT-DEPENDENT SHORT HYPOCOTYLS 1)
At5g34930	AT5G34930	0.00912	arogenate dehydrogenase

Development • Supplementary information

At5g37950	AT5G37950	0.00619	UDP-glycosyltransferase-like protein /// UDP-glycosyltransferase 76E9
At5g39030	AT5G39030	0.00686	putative receptor-like protein kinase
At5g39220	AT5G39220	0.00047	hydrolase, alpha/beta fold family protein
At5g40830	AT5G40830	0.0014	S-adenosyl-L-methionine-dependent methyltransferases superfamily protein
At5g41600	BTI3	0.0019	BTI3 (VIRB2-INTERACTING PROTEIN 3)
At5g42500	AT5G42500	0	disease resistance-responsive (dirigent-like protein) family protein
At5g43330	AT5G43330	0.00036	malate dehydrogenase
At5g44750	REV1	0.00648	REV1; DNA-directed DNA polymerase
At5g45010	ATDSS1(V)	0.00234	ATDSS1(V) (Arabidopsis dss1 homolog on chromosome V)
At5g46140	AT5G46140	0.00149	hypothetical protein
At5g47550	AT5G47550	1.4E-05	cysteine proteinase inhibitor 5
At5g47720	AT5G47720	0.00479	acetoacetyl-CoA thiolase 1
At5g47920	AT5G47920	0.00217	hypothetical protein
At5g47980	AT5G47980	0.00596	putative BAHD acyltransferase
At5g49480	ATCP1	0.00779	ATCP1 (Ca2+-binding protein 1); calcium ion binding
At5g49610	AT5G49610	0.00017	F-box protein
At5g49630	AAP6	0.00101	AAP6 (AMINO ACID PERMEASE 6); acidic amino acid transmembrane transporter
At5g50800	AtSWEET13	0	bidirectional sugar transporter SWEET13
At5g50970	AT5G50970	0.00455	transducin/WD40 domain-containing protein
At5g52180	AT5G52180	0.00413	hypothetical protein
At5g52860	AT5G52860	0.00087	ABC transporter G family member 8
At5g53660	AtGRF7	0.00036	AtGRF7 (GROWTH-REGULATING FACTOR 7); transcription activator
At5g54510	DFL1	0.00969	DFL1 (DWARF IN LIGHT 1); indole-3-acetic acid amido synthetase
At5g54840	SGP1	7.1E-06	SGP1; GTP binding
At5g55450	AT5G55450	0.00848	bifunctional inhibitor/lipid-transfer protein/seed storage 2S albumin-like protein
At5g57530	XTH12	0.00523	probable xyloglucan endotransglucosylase/hydrolase protein 12
At5g58390	AT5G58390	0	peroxidase
At5g58500	LSH5	0.00925	LSH5 (LIGHT SENSITIVE HYPOCOTYLS 5)
At5g58860	CYP86A1	0.00916	CYP86A1 (CYTOCHROME P450 86 A1); fatty acid (omega-1)-hydroxylase/ oxygen binding
At5g60490	FLA12	0.00735	FLA12
At5g61290	AT5G61290	0.00069	flavin-containing monooxygenase FMO GS-OX-like 8
At5g61660	AT5G61660	4.1E-05	glycine-rich protein
At5g62680	AT5G62680	4.6E-07	glucosinolate transporter-2
At5g63320	NPX1	0.00429	nuclear protein X1; ABA response
At5g63420	emb2746	0.00568	emb2746 (embryo defective 2746); DNA binding / catalytic/ hydrolase

At5g64280	DIT2.2	0.00439	DIT2.2 (dicarboxylate transporter 2.2); oxoglutarate:malate antiporter
At5g64570	XYL4	0.00021	XYL4; hydrolase, hydrolyzing O-glycosyl compounds / xylan 1,4-beta-xylosidase
At5g64740	CESA6	0.00235	CESA6 (CELLULOSE SYNTHASE 6); cellulose synthase/ transferase, transferring glycosyl groups
At5g65020	ANNAT2	0.00411	ANNAT2 (Annexin Arabidopsis 2); calcium ion binding / calcium-dependent phospholipid binding
At5g65140	TPPJ	0.00445	probable trehalose-phosphate phosphatase J
At5g65410	HB25	0.00217	HB25 (HOMEOBOX PROTEIN 25); DNA binding / sequence-specific DNA binding / transcription factor/ transcription regulator
At5g65510	AIL7	5.7E-06	AIL7 (AINTEGUMENTA-like 7); DNA binding / transcription factor
At5g66390	AT5G66390	0.00784	peroxidase
At5g66730	AT5G66730	0.00099	C2H2-like zinc finger protein
At5g67300	MYB44	0.00013	MYB44 (MYB DOMAIN PROTEIN 44; MYB domain protein R1); DNA binding / transcription factor
At5g67600	AT5G67600	0.00146	hypothetical protein

Table S4

List of 465 DEGs identified in the meta-analysis of STMoe 8h, STMoe 24h, STMRNAi 72h and STMRNAi 9d datasets. Locus ID, gene name, meta-analysis Q value and a general description are shown.

Locus ID	Gene Name	Q Value	Description
At1g05710	AT1G05710	0.000161244	basic helix-loop-helix domain-containing protein
At1g07640	OBP2	0.00062352	OBP2; DOF transcription factor
At1g09530	PAP3	0.009945263	PAP3 (PHYTOCHROME ASSOCIATED PROTEIN 3)/ PIF3; transcription factor
At1g13400	JGL	0.001799741	JGL/ NUB (NUBBIN); nucleic acid binding / zinc ion binding
At1g13600	AtbZIP58	0.000889281	AtbZIP58 (Arabidopsis thaliana basic leucine-zipper 58); transcription factor
At1g15920	AT1G15920	0.008457093	polynucleotidyl transferase, ribonuclease H-like superfamily protein
At1g30650	WRKY14	0	WRKY14; transcription factor
At1g53230	TCP3	0.003653445	TCP3; transcription factor
At1g62360	STM	0	STM (SHOOT MERISTEMLESS); transcription factor
At1g70510	KNAT2	0.001040801	KNAT2 (KNOTTED-LIKE FROM ARABIDOPSIS THALIANA 2); transcription factor
At1g74500	TMO7	0.000407314	transcription factor bHLH135, AtBS1, TMO7
At1g74890	ARR15	0.00600655	ARR15 (RESPONSE REGULATOR 15); transcription regulator/ two-component response regulator
At1g75390	AtbZIP44	0.000414656	AtbZIP44 (Arabidopsis thaliana basic leucine-zipper 44); transcription factor
At1g76420	CUC3	0	CUC3 (CUP SHAPED COTYLEDON3); transcription factor
At1g78080	RAP2.4	0.008181263	RAP2.4 (related to AP2 4); transcription factor
At1g79000	HAC1	0.009524288	HAC1 (HISTONE ACETYLTRANSFERASE OF THE CBP FAMILY 1); transcription cofactor
At2g01830	WOL	0.0000866	WOL (WOODEN LEG); cytokinin receptor
At2g21650	MEE3	0.000414656	MEE3 (MATERNAL EFFECT EMBRYO ARREST 3); transcription factor
At2g26580	YAB5	0.000414041	YAB5 (YABBY5); transcription factor
At2g27990	BLH8	0	BLH8 (BEL1-LIKE HOMEODOMAIN 8); transcription factor
At2g28510	AT2G28510	0.000880453	Dof zinc finger protein DOF2.1
At2g34830	WRKY35	0.000743471	WRKY35 (WRKY DNA-binding protein 35); transcription factor
At2g37630	AS1	0.000200557	AS1 (ASYMMETRIC LEAVES 1); transcription factor
At2g41370	BOP2	0	BOP2 (BLADE ON PETIOLE2); protein binding
At2g41940	ZFP8	0.0000501	ZFP8 (ZINC FINGER PROTEIN 8); transcription factor
At2g46310	CRF5	0.009255613	CRF5 (CYTOKININ RESPONSE FACTOR 5); DNA binding / transcription factor
At2g46590	DAG2	0.000316787	DAG2 (DOF AFFECTING GERMINATION 2); transcription factor
At3g05800	AIF1	0.006563811	activation-tagged BRI1 suppressor 1-interacting factor 1
At3g15030	TCP4	0.0000925	TCP4 (TCP family transcription factor 4); transcription factor
At3g15170	CUC1	0	CUC1 (CUP-SHAPED COTYLEDON1); transcription factor
At3g24340	CHR40	0	chr40 (chromatin remodeling 40); ATP binding / DNA binding / helicase/ nucleic acid binding
At3g24490	AT3G24490	0.002979148	alcohol dehydrogenase transcription factor myb/SANT-like protein
At3g25940	AT3G25940	0.006955961	TFIIB zinc-binding protein
At3g27650	LBD25	0	LBD25 (LOB DOMAIN-CONTAINING PROTEIN 25)
At3g27810	ATMYB21	0	ATMYB21 (ARABIDOPSIS THALIANA MYB DOMAIN PROTEIN 21); transcription factor
At3g48550	AT3G48550	0.001172729	C2H2-like zinc-finger protein
At3g50700	AtIDD2	0.000363783	AtIDD2 (Arabidopsis thaliana Indeterminate(ID)-Domain 2); transcription factor
At3g54620	BZIP25	0.004660563	BZIP25 (BASIC LEUCINE ZIPPER 25); transcription factor
At3g57130	BOP1	0.000125354	BOP1 (BLADE ON PETIOLE 1); protein binding
At3g60800	AT3G60800	0.002558158	DHHC-type zinc finger family protein/ protein acyltransferase
At3g61250	AtMYB17	0.000201657	AtMYB17 (myb domain protein 17); transcription factor
At4g08150	KNAT1	0.000247902	KNAT1 (KNOTTED-LIKE FROM ARABIDOPSIS THALIANA1)/ BREVIPEDICELLUS (BP) transcription factor
At4g31805	POLAR	0.0006154	POLAR; transcription factor
At4g34400	AT4G34400	0.002284601	AP2/B3-like transcriptional factor family protein
At4g35040	bZIP19	0.008396916	transcription factor bZIP19
At4g37650	SHR	0.000722644	SHR (SHORT ROOT); transcription factor
At5g01840	OFP1	0	OFP1 (OVATE FAMILY PROTEIN 1); transcription factor
At5g07690	ATMYB29	0.007035545	ATMYB29 (ARABIDOPSIS THALIANA MYB DOMAIN PROTEIN 29); transcription factor
At5g10140	FLC	0.00667501	FLC (FLOWERING LOCUS C)/ AGL25; transcription factor
At5g15840	CO	0.001530423	CO (CONSTANS); transcription factor
At5g16600	MYB43	0.001110735	MYB43 (myb domain protein 43); transcription factor
At5g22650	HD2B	0.000277916	HD2B (HISTONE DEACETYLASE 2B); histone deacetylase
At5g53660	AtGRF7	0.000363838	AtGRF7 (GROWTH-REGULATING FACTOR 7); transcription activator
At5g65410	HB25	0.002174398	HB25 (HOMEOBOX PROTEIN 25); transcription factor
At5g65510	AIL7	0.00000573	AIL7 (AINTEGUMENTA-like 7); transcription factor
At5g66730	IDD1	0.000990016	C2H2-like zinc finger protein
At5g67300	ATMYB44	0.000125321	MYBR1 (MYB DOMAIN PROTEIN R1); AtMYB44; transcription factor

Table S5.

List of 57 genes (mostly transcription factors) used in network construction. Locus ID, gene name, Q-value and descriptions are shown.

Development • Supplementary information

Locus ID	Gene Name	Description	adjPVal	logFC
**Auxin**				
At2g45210	AT2G45210	SAUR-like auxin-responsive protein	3.32E-15	2.616
At1g16510	AT1G16510	SAUR-like auxin-responsive protein	7.587E-18	2.373
At2g33830	AT2G33830	dormancy/auxin associated protein	1.245E-07	2.342
At2g46690	AT2G46690	SAUR-like auxin-responsive protein	1.304E-18	2.105
At1g43040	AT1G43040	putative auxin-responsive protein	3.416E-05	1.704
At1g62500	AT1G62500	bifunctional inhibitor/lipid-transfer protein/seed storage 2S albumin superfamily protein	2.75E-13	1.498
At1g48660	AT1G48660	auxin-responsive GH3-like protein	1.867E-10	1.442
At3g12830	AT3G12830	SAUR-like auxin-responsive protein	1.467E-10	1.406
At4g34750	AT4G34750	SAUR-like auxin-responsive family protein	3.962E-12	1.297
At4g02380	SAG21	SAG21 (SENESCENCE-ASSOCIATED GENE 21)	2.224E-05	1.275
At1g75580	AT1G75580	SAUR-like auxin-responsive protein	3.291E-10	1.190
At5g13360	AT5G13360	auxin-responsive GH3 family protein /// auxin-responsive GH3 family protein	2.615E-09	1.079
At5g50760	AT5G50760	SAUR-like auxin-responsive protein	0.0004799	1.077
At5g35735	AT5G35735	putative auxin-responsive protein	4.671E-07	0.905
At4g34770	AT4G34770	SAUR-like auxin-responsive protein	0.0010311	0.849
At5g60450	ARF4	ARF4 (AUXIN RESPONSE FACTOR 4); transcription factor	1.372E-06	0.780
At2g38120	AUX1	AUX1 (AUXIN RESISTANT 1); amino acid transmembrane transporter/ auxin binding / auxin influx transmembrane transporter/ transporter	3.293E-07	0.758
At1g28130	GH3.17	GH3.17; indole-3-acetic acid amido synthetase	7.015E-06	0.729
At1g56150	AT1G56150	SAUR-like auxin-responsive protein	0.0016192	0.657
At1g56220	AT1G56220	dormancy/auxin associated protein	0.000265	0.630
At1g77690	LAX3	LAX3 (LIKE AUX1 3); amino acid transmembrane transporter/ auxin influx transmembrane transporter/ transporter	2.81E-05	0.601
At1g60680	AT1G60680	probable aldo-keto reductase 2	0.0015325	0.597
At2g33860	ETT	ETT (ETTIN); DNA binding / transcription factor	5.365E-06	0.543
At2g28350	ARF10	ARF10 (AUXIN RESPONSE FACTOR 10); miRNA binding / transcription factor	4.303E-07	0.539
At1g75590	AT1G75590	SAUR-like auxin-responsive protein	0.0012751	0.424
At1g58100	AT1G58100	transcription factor TCP8	5.5E-07	0.414
At1g80680	SAR3	SAR3 (SUPPRESSOR OF AUXIN RESISTANCE 3); porin	4.937E-05	0.408
At1g19220	ARF19	ARF19 (AUXIN RESPONSE FACTOR 19); DNA binding / transcription factor	0.0001852	0.405
At3g26810	AFB2	AFB2 (AUXIN SIGNALING F-BOX 2); auxin binding / ubiquitin-protein ligase	2.93E-05	0.401
At1g05180	AXR1	AXR1 (AUXIN RESISTANT 1); small protein activating enzyme	1.881E-06	0.376
At1g30330	ARF6	ARF6 (AUXIN RESPONSE FACTOR 6); transcription factor	0.0005259	0.374
At1g73590	PIN1	PIN1 (PIN-FORMED 1); transporter	0.0041295	0.371
At2g36910	ABCB1	ABCB1 (ATP BINDING CASSETTE SUBFAMILY B1); ATPase, auxin efflux transmembrane transporter/ calmodulin binding	0.0013574	0.365

Development • Supplementary information

At4g03190	GRH1	GRH1 (GRR1-LIKE PROTEIN 1); auxin binding / protein binding / ubiquitin-protein ligase	0.0030703	0.365
At3g28860	ABCB19	ABCB19; ATPase, coupled to transmembrane movement of substances / auxin efflux transmembrane transporter	0.0038487	0.364
At2g16580	AT2G16580	SAUR-like auxin-responsive protein	0.0057893	0.341
At1g33410	SAR1	SAR1 (suppressor of auxin resistance1)	0.004734	0.326
At4g02980	ABP1	ABP1 (ENDOPLASMIC RETICULUM AUXIN BINDING PROTEIN 1); auxin binding	0.0020896	0.290
At5g64600	AT5G64600	O-fucosyltransferase family protein	0.0034822	0.250
At2g22670	IAA8	IAA8; transcription factor	0.0012309	-0.239
At3g25880	AT3G25880	hypothetical protein	0.0012502	-0.298
At2g21200	AT2G21200	SAUR-like auxin-responsive protein	0.0067753	-0.308
At1g53700	WAG1	WAG1 (WAG 1); kinase/ protein serine/threonine kinase	0.0084228	-0.316
At1g59750	ARF1	ARF1 (AUXIN RESPONSE FACTOR 1); DNA binding / transcription factor	0.000588	-0.319
At2g24400	AT2G24400	SAUR-like auxin-responsive protein	0.0023434	-0.330
At2g18010	AT2G18010	SAUR-like auxin-responsive protein	0.0050976	-0.334
At5g63390	AT5G63390	O-fucosyltransferase family protein	0.0010621	-0.349
At1g29450	AT1G29450	SAUR-like auxin-responsive protein	0.0005803	-0.389
At5g01990	AT5G01990	auxin efflux carrier family protein	1.962E-06	-0.413
At3g03840	AT3G03840	SAUR-like auxin-responsive protein	0.001322	-0.442
At5g43700	ATAUX2-11	ATAUX2-11 (AUXIN INDUCIBLE 2-11); DNA binding / transcription factor	0.0002702	-0.505
At5g01240	LAX1	auxin transporter-like protein 1	7.847E-07	-0.521
At1g34310	ARF12	ARF12 (AUXIN RESPONSE FACTOR 12); transcription factor	1.026E-05	-0.556
At4g28640	IAA11	IAA11 (INDOLE-3-ACETIC ACID INDUCIBLE 11); transcription factor	2.091E-06	-0.581
At3g15540	IAA19	IAA19 (INDOLE-3-ACETIC ACID INDUCIBLE 19); transcription factor	3.951E-06	-0.620
At1g23740	AT1G23740	alkenal/one oxidoreductase	4.506E-07	-0.629
At2g21050	LAX2	like AUXIN RESISTANT 2	4.493E-06	-0.647
At2g33310	IAA13	IAA13; transcription factor	3.715E-10	-0.665
At5g62010	ARF2	ARF2 (AUXIN RESPONSE FACTOR 2); protein binding / transcription factor	1.938E-06	-0.703
At1g60690	AT1G60690	NAD(P)-linked oxidoreductase superfamily protein	7.103E-10	-0.713
At1g76530	AT1G76530	auxin efflux carrier family protein	1.584E-05	-0.714
At1g23080	PIN7	PIN7 (PIN-FORMED 7); auxin efflux transmembrane transporter/ auxin:hydrogen symporter/ transporter	6.382E-10	-0.770
At1g15580	IAA5	IAA5 (INDOLE-3-ACETIC ACID INDUCIBLE 5); transcription factor	1.301E-07	-0.776
At4g38850	SAUR15	SAUR15 (SMALL AUXIN UPREGULATED 15)	3.276E-05	-0.850
At3g23030	IAA2	IAA2 (INDOLE-3-ACETIC ACID INDUCIBLE 2); transcription factor	1.181E-07	-0.939
At5g13320	PBS3	PBS3 (AVRPPHB SUSCEPTIBLE 3)	1.619E-05	-0.957
At4g38840	AT4G38840	SAUR-like auxin-responsive protein	1.969E-07	-0.975
At5g62000	ARF2	ARF2 (AUXIN RESPONSE FACTOR 2); protein binding / transcription factor	2.578E-05	-0.976

Development • Supplementary information

At1g70940	PIN3	PIN3 (PIN-FORMED 3); auxin:hydrogen symporter/ transporter	1.463E-10	-1.029
At1g29510	SAUR68	SAUR68 (SMALL AUXIN UPREGULATED 68)	0.0003599	-1.163
At2g04850	AT2G04850	auxin-responsive family protein	3.088E-14	-1.166
At5g08330	AT5G08330	transcription factor TCP21	3.83E-11	-1.169
At4g34760	AT4G34760	SAUR-like auxin-responsive protein 9	8.296E-10	-1.191
At1g29460	AT1G29460	SAUR-like auxin-responsive protein	2.289E-05	-1.206
At1g04240	SHY2	SHY2 (SHORT HYPOCOTYL 2); transcription factor	3.185E-12	-1.278
At2g28085	AT2G28085	SAUR-like auxin-responsive protein	5.062E-10	-1.329
At4g14560	IAA1	IAA1 (INDOLE-3-ACETIC ACID INDUCIBLE); protein binding / transcription factor	1.422E-11	-1.336
At1g04250	AXR3	AXR3 (AUXIN RESISTANT 3); transcription factor	6.298E-13	-1.355
At2g46530	ARF11	ARF11 (AUXIN RESPONSE FACTOR 11); transcription factor	5.437E-10	-1.456
At5g18060	AT5G18060	SAUR-like auxin-responsive protein	1.784E-06	-1.472
At1g29500	AT1G29500	SAUR-like auxin-responsive protein	2.68E-07	-1.640
At4g38860	AT4G38860	SAUR-like auxin-responsive protein	3.873E-08	-1.744
At2g01420	PIN4	PIN4 (PIN-FORMED 4); auxin:hydrogen symporter/ transporter	1.244E-16	-1.823
At1g29440	AT1G29440	SAUR-like auxin-responsive protein	1.612E-06	-1.887
At4g12980	AT4G12980	putative auxin-responsive protein	3.909E-14	-2.199
At1g29430	AT1G29430	SAUR-like auxin-responsive protein /// SAUR-like auxin-responsive protein	1.55E-15	-3.877
**GA**				
At4g09600	GASA3	GASA3 (GAST1 PROTEIN HOMOLOG 3)	5.464E-16	2.345
At5g07200	GA20OX3	YAP169; gibberellin 20-oxidase	9.286E-17	2.189
At1g30040	ATGA2OX2	ATGA2OX2 (GIBBERELLIN 2-OXIDASE); gibberellin 2-beta-dioxygenase	2.736E-10	0.946
At3g46500	AT3G46500	oxidoreductase, 2OG-Fe(II) oxygenase family protein	6.054E-08	0.684
At5g14920	AT5G14920	gibberellin-regulated protein	0.0002399	0.660
At2g14900	AT2G14900	gibberellin-regulated protein	0.0007627	-0.509
At5g51310	AT5G51310	2-oxoglutarate (2OG) and Fe(II)-dependent oxygenase superfamily protein	1.519E-05	-0.731
At1g52800	AT1G52800	oxidoreductase, 2OG-Fe(II) oxygenase family protein	9.433E-07	-0.779
At5g59845	AT5G59845	gibberellin-regulated protein	4.669E-05	-0.989
At1g66350	RGL1	RGL1 (RGA-LIKE 1); transcription factor	5.903E-11	-1.009
At4g25420	GA20OX1	GA20OX1; gibberellin 20-oxidase	3.765E-09	-1.125
At1g74670	AT1G74670	gibberellin-regulated protein 6	0.0002708	-1.284
At4g26420	GAMT1	GAMT1; S-adenosylmethionine-dependent methyltransferase/ gibberellin carboxyl-O-methyltransferase	8.752E-13	-1.360
At1g22690	AT1G22690	gibberellin-regulated protein 9	2.062E-05	-1.441
At3g46490	AT3G46490	iron ion binding / oxidoreductase/ oxidoreductase protein	1.554E-22	-3.587

**Cytokinin**				
At3g63110	ATIPT3	ATIPT3 (ARABIDOPSIS THALIANA ISOPENTENYLTRANSFERASE 3); ATP binding / tRNA isopentenyltransferase/ transferase, transferring alkyl or aryl (other than methyl) groups	1.998E-20	1.898
At3g48100	ARR5	ARR5 (ARABIDOPSIS RESPONSE REGULATOR 5); transcription regulator/ two-component response regulator	1.436E-10	1.429
At5g05860	UGT76C2	UGT76C2; UDP-glycosyltransferase/ cis-zeatin O-beta-D-glucosyltransferase/ cytokinin 7-beta-glucosyltransferase/ cytokinin 9-beta-glucosyltransferase/ trans-zeatin O-beta-D-	7.896E-09	1.087
At5g62920	ARR6	ARR6 (RESPONSE REGULATOR 6); transcription regulator/ two-component response regulator	2.603E-07	1.080
At3g57040	ARR9	ARR9 (RESPONSE REGULATOR 9); transcription regulator/ two-component response regulator	8.878E-08	1.059
At3g61630	CRF6	CRF6 (CYTOKININ RESPONSE FACTOR 6); DNA binding / transcription factor	8.735E-08	1.031
At2g01830	WOL	WOL (WOODEN LEG); cytokinin receptor/ osmosensor/ phosphoprotein phosphatase/ protein histidine kinase	1.608E-06	0.848
At4g31920	ARR10	ARR10 (ARABIDOPSIS RESPONSE REGULATOR 10); transcription factor/ two-component response regulator	4.466E-10	0.800
At4g18020	APRR2	APRR2; transcription factor/ two-component response regulator	1.832E-06	0.470
At5g53290	CRF3	CRF3 (CYTOKININ RESPONSE FACTOR 3); DNA binding / transcription factor	0.0019412	0.442
At3g23630	IPT7	ATIPT7; ATP binding / tRNA isopentenyltransferase/ transferase, transferring alkyl or aryl (other than methyl) groups	0.0014064	0.42656
At5g19040	IPT5	IPT5; ATP binding / tRNA isopentenyltransferase/ transferase, transferring alkyl or aryl (other than methyl) groups	0.0005782	0.412
At2g41510	CKX1	CKX1 (CYTOKININ OXIDASE/DEHYDROGENASE 1); cytokinin dehydrogenase	0.0069579	-0.367
At4g29740	CKX4	CKX4 (CYTOKININ OXIDASE 4); amine oxidase/ cytokinin dehydrogenase	0.000638	-0.507
At4g11140	CRF1	CRF1 (CYTOKININ RESPONSE FACTOR 1); DNA binding / transcription factor	9.313E-06	-0.515
At5g06300	AT5G06300	cytokinin riboside 5'-monophosphate phosphoribohydrolase LOG7	2.879E-05	-0.517
At5g60100	APRR3	APRR3 (ARABIDOPSIS PSEUDO-RESPONSE REGULATOR 3); transcription regulator/ two-component response regulator	1.919E-05	-0.568
At2g46790	APRR9	APRR9 (ARABIDOPSIS PSEUDO-RESPONSE REGULATOR 9); protein binding / transcription regulator/ two-component response regulator	0.007484	-0.644
At3g63440	CKX6	CKX6 (CYTOKININ OXIDASE/DEHYDROGENASE 6); cytokinin dehydrogenase	3.248E-09	-0.916
At4g23750	CRF2	CRF2 (CYTOKININ RESPONSE FACTOR 2); DNA binding / transcription factor	3.425E-11	-1.051
At5g11950	AT5G11950	cytokinin riboside 5'-monophosphate phosphoribohydrolase LOG8	5.074E-12	-1.190
At2g40670	ARR16	ARR16 (ARABIDOPSIS RESPONSE REGULATOR 16); transcription regulator/ two-component response regulator	6.034E-11	-1.907
At2g28305	LOG1	cytokinin riboside 5'-monophosphate phosphoribohydrolase LOG1	2.459E-15	-2.208

Table S6.

Table of DEGs relating to auxin, GA and cytokinin in the long-term induced STMoe 9d dataset. Locus ID, Gene name, description, adjusted p-value and log_2 fold-change are shown.

Development • Supplementary information

LOCUS ID	Gene Name	Adj Pval	LogFC
**AIL/ PLT**			
At5g10510	AIL6/ PLT3	4.21E-12	**1.381493**
At5g65510	AIL7/ PLT7	4.97E-21	**2.678361**
At4g37750	ANT	1.17E-06	**0.784359**
**CUC**			
At3g15170	CUC1	3.56E-18	**2.258847**
At5g53950	CUC2	3.19E-06	**0.671852**
At1g76420	CUC3	1.5E-16	**1.981847**
**TCP class-1**			
At1g35560	TCP23	6.39E-05	**0.447153**
At1g58100	TCP8	5.5E-07	**0.414081**
At1g69690	TCP15	1.12E-08	**-0.86006**
At2g45680	TCP9	6.19E-07	**-0.82307**
At5g08330	TCP21	3.83E-11	**-1.16858**
At5g51910	TCP19	0.008529	**0.345003**
**TCP class-2**			
At1g30210	TCP24	2.42E-12	**-0.91314**
At1g53230	TCP3	1.04E-22	**-3.40637**
At2g31070	TCP10	4.5E-21	**-1.81937**
At3g15030	TCP4	4.61E-19	**-2.7369**
At4g18390	TCP2	2.21E-07	**-0.61843**
At5g08070	TCP17	0.000328	**-0.54822**
At5g60970	TCP5	4.22E-06	**-0.54439**
**AS2/ LOB**			
At1g65620	AS2	9.24E-10	**0.865133**
At1g31320	LBD4	0.000634	**0.472297**
At1g67100	LBD40	2.11E-05	**0.801955**
At2g40470	LBD15	0.000293	**-0.4265**
At2g42430	LBD16	0.001515	**0.431179**
At3g02550	LBD41	6.81E-10	**2.942522**
At3g27650	LBD25	3.26E-11	**0.94743**
At3g49940	LBD38	2.9E-14	**1.889533**
At4g37540	LBD39	1.8E-12	**1.998316**
**AS1**			
At2g37630	AS1	5.41E-06	**0.488353**
**LSH**			
At1g07090	LSH6	4.89E-09	**0.755579**
At1g78815	LSH7	1.45E-05	**0.597886**
At2g31160	LSH3	1.66E-19	**2.068311**
At2g42610	LSH10	4.44E-12	**1.142444**
At3g04510	LSH2	0.001493	**0.496277**
At3g23290	LSH4	1.72E-22	**2.880061**
At5g58500	LSH5	8.7E-11	**1.071909**

Table S7.

Table of genes encoding transcription factors belonging to the AIL7/PLT, CUC, TCP, AS2/LOB and LSH families in the long-term induced STMoe 9d dataset. Locus ID, gene name, adjusted p-value and log_2 fold-change are shown.

Development • Supplementary information

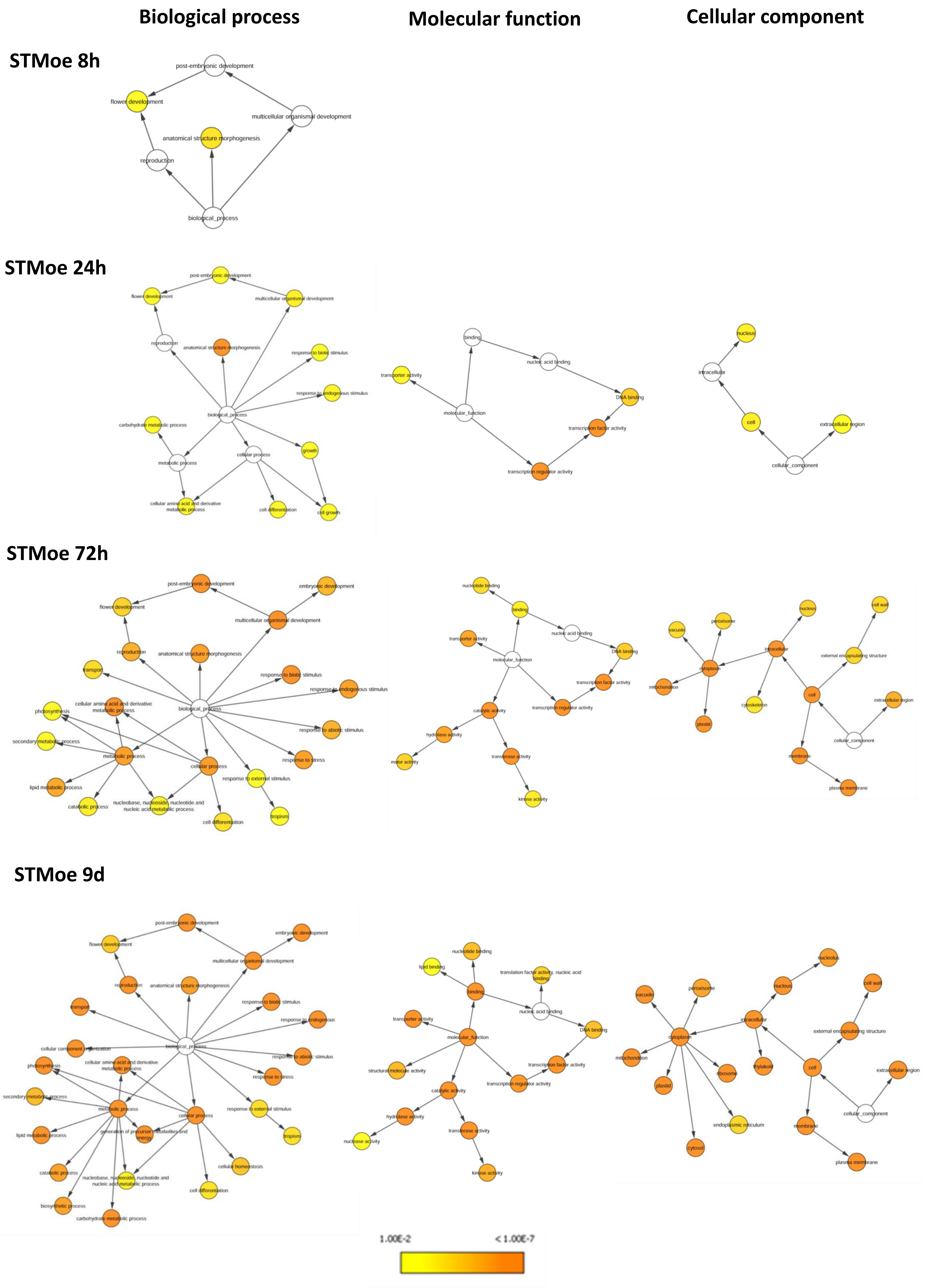

Development • Supplementary information

Figure S1.

Gene ontology (GO) SLIM enrichment analysis of DEGs identified at each STMoe time-point. Performed with BINGO using hypergeometric test, $p<0.05$ significance threshold and Benjamini and Hochberg FDR correction. GO slim plants annotation set was used. All nodes were set to the same size for clarity. Progressive enrichment of GO categories is evident across the time-course for biological process, molecular function and cellular component. No significant enrichment was found for molecular function or cellular component at the 8h STMoe time-point.

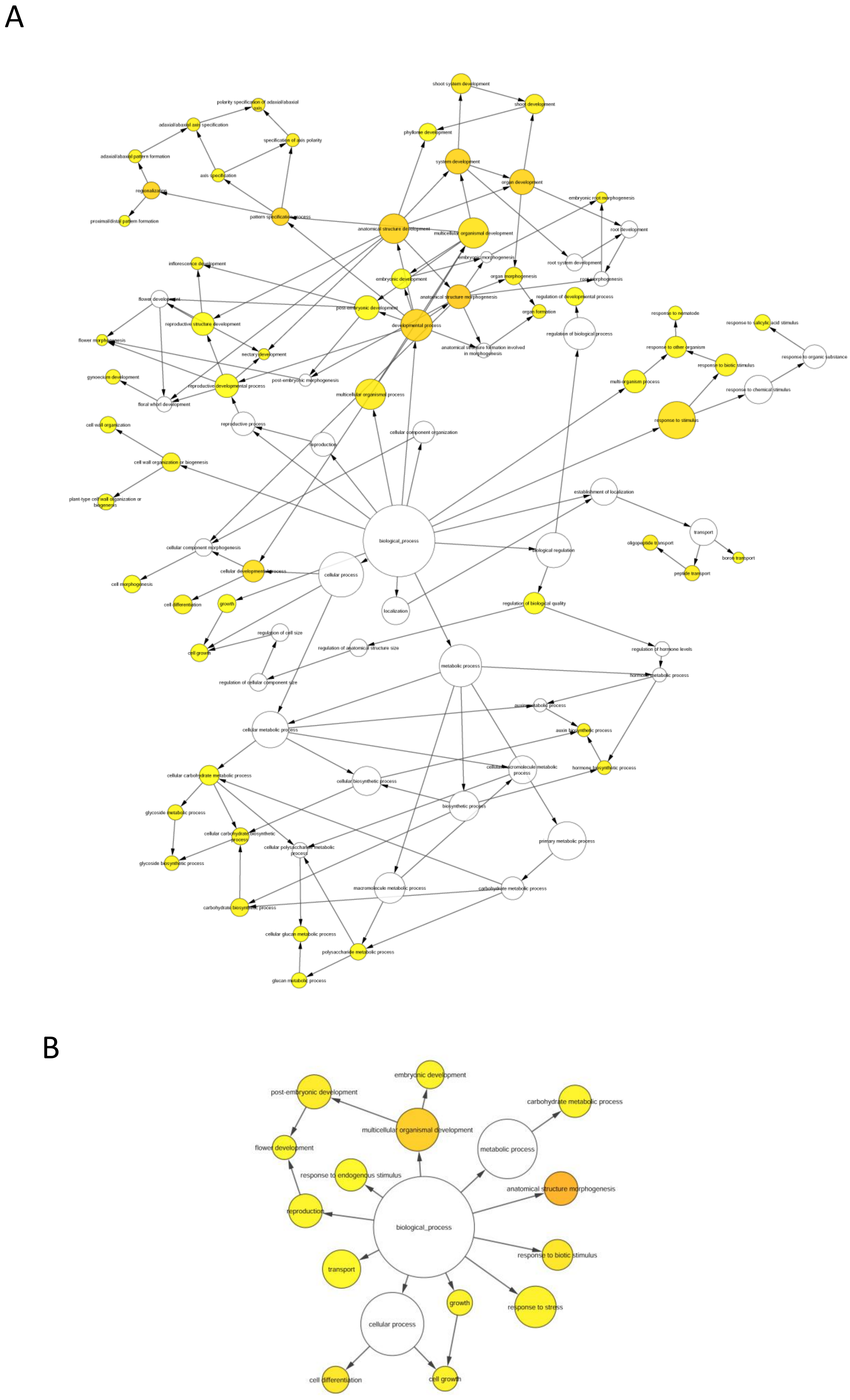

Development • Supplementary information

C

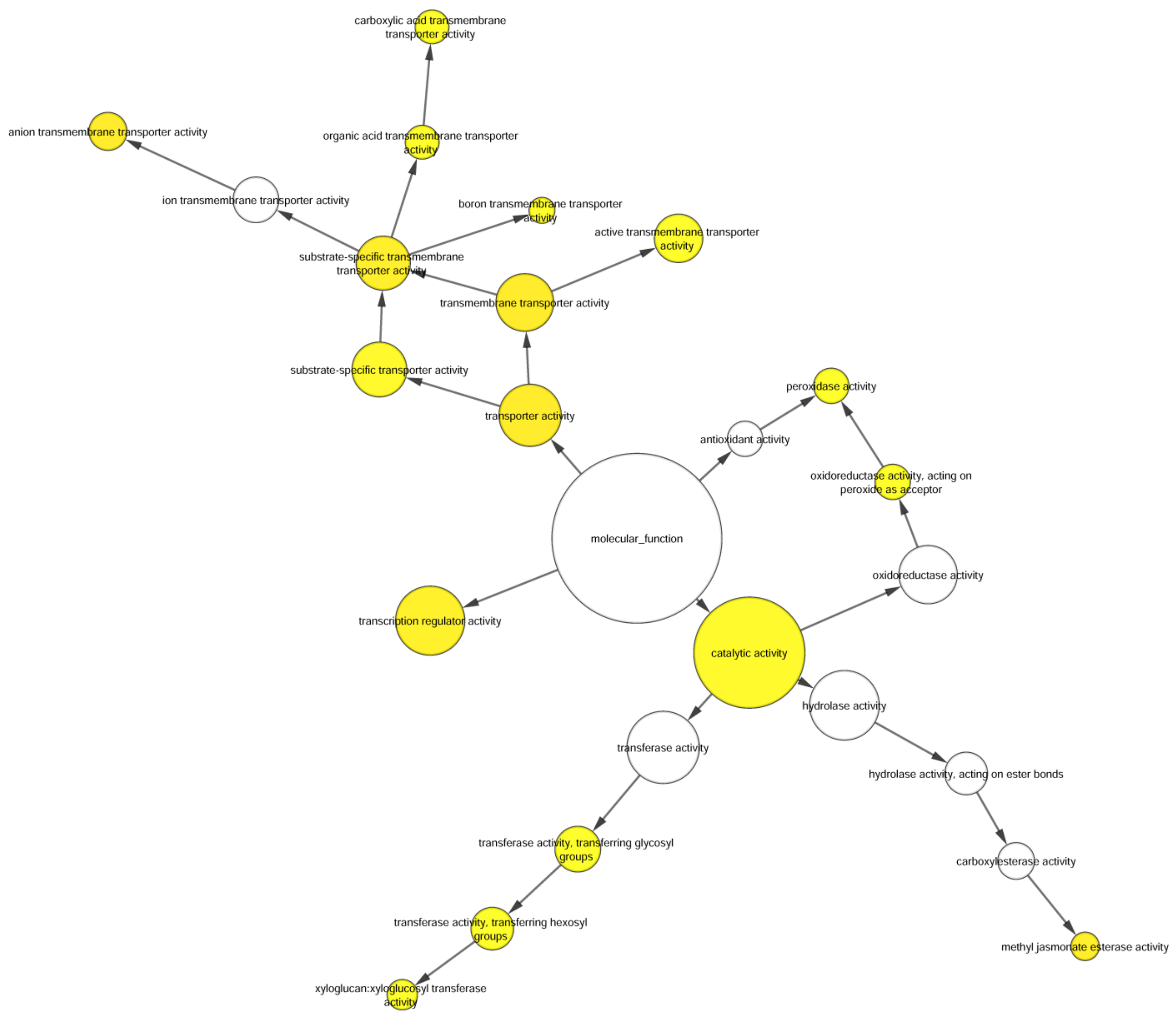

D

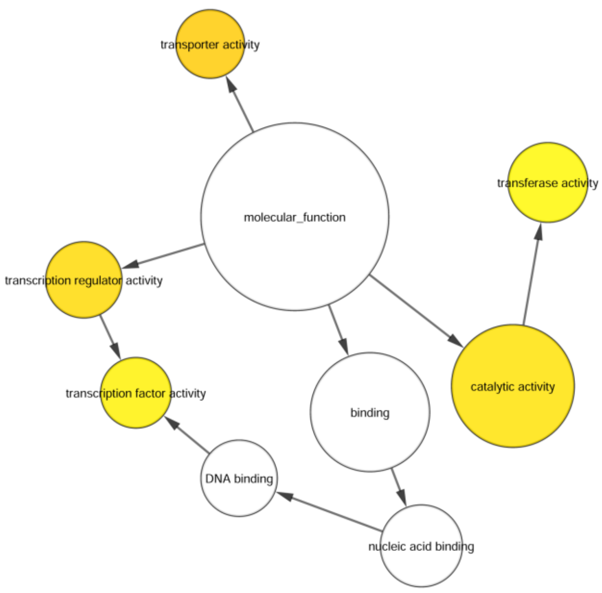

Development • Supplementary information

E

LOCUS ID	Gene name	q-value	Description
**Cytokinin**			
At1g74890	ARR15	0.00601	ARR15 (RESPONSE REGULATOR 15); transcription regulator/ two-component response regulator
At2g01830	WOL	8.7E-05	WOL (WOODEN LEG); cytokinin receptor/ osmosensor/ phosphoprotein phosphatase/ protein histidine kinase
At2g46310	CRF5	0.00926	CRF5 (CYTOKININ RESPONSE FACTOR 5); AP2 domain DNA binding / transcription factor
**GA**			
At5g01840	OFP1	0	OFP1 (OVATE FAMILY PROTEIN 1); protein binding / transcription repressor
At5g65410	HB25	0.00217	HB25 (HOMEOBOX PROTEIN 25); DNA binding / sequence-specific DNA binding / transcription factor/ transcription regulator
**Auxin**			
At1g08980	AMI1	0.00052	AMI1 (AMIDASE 1); amidase/ hydrolase, acting on carbon-nitrogen (but not peptide) bonds / indoleacetamide hydrolase
At1g58360	AAP1	0.003	AAP1 (AMINO ACID PERMEASE 1); amino acid transmembrane transporter/ neutral amino acid transmembrane transporter
At1g70560	TAA1	9.5E-06	TAA1 (TRYPTOPHAN AMINOTRANSFERASE OF ARABIDOPSIS 1);
At5g11320	YUC4	0.00036	YUC4 (YUCCA4); flavinoid monooxygenase/ oxidoreductase
At5g16530	PIN5	0.00251	PIN5 (PIN-FORMED 5); auxin:hydrogen symporter/ transporter
At5g20960	AAO1	0.0008	AAO1 (ARABIDOPSIS ALDEHYDE OXIDASE 1); aldehyde oxidase/ indole-3-acetaldehyde oxidase
At5g23810	AAP7	0	AAP7; amino acid transmembrane transporter
At5g49630	AAP6	0.00101	AAP6 (AMINO ACID PERMEASE 6); acidic amino acid transmembrane transporter
**Cell wall**			
At1g05570	CALS1	0.00375	CALS1 (CALLOSE SYNTHASE 1); 1,3-beta-glucan synthase/ transferase, transferring glycosyl groups
At1g06490	CALS7/ ATGSL07	0.00131	ATGSL07/ CALS7 (glucan synthase-like 7); 1,3-beta-glucan synthase/ transferase, transferring glycosyl groups
At1g20190	ATEXPA11	1.9E-07	ATEXPA11 (ARABIDOPSIS THALIANA EXPANSIN 11)
At1g56710	AT1G56710	8.9E-06	polygalacturonase; pectin lyase-like protein
At1g61810	BGLU45	0	BGLU45 (BETA-GLUCOSIDASE 45); catalytic/ cation binding / hydrolase, hydrolyzing O-glycosyl compounds
At1g66280	BGLU22	0.00198	BGLU22; catalytic/ cation binding / hydrolase, hydrolyzing O-glycosyl compounds
At2g36710	AT2G36710	0.00534	putative pectinesterase 15
At2g36870	XTH32	0.00125	probable xyloglucan endotransglucosylase/hydrolase protein 32
At2g37640	EXP3	0.0017	EXPANSIN3
At2g38080	IRX12	0.0047	IRX12 (IRREGULAR XYLEM 12); laccase
At2g47550	AT2G47550	0.00047	probable pectinesterase/pectinesterase inhibitor 20
At3g23730	XTH16	0.00027	xyloglucan endotransglucosylase/hydrolase protein 16
At3g59010	PME61	0.00169	pectinesterase 35
At3g62740	BGLU7	0	BGLU7 (BETA GLUCOSIDASE 7); catalytic/ cation binding / hydrolase, hydrolyzing O-glycosyl compounds
At4g17030	ATEXLB1	2.3E-05	ATEXLB1 (ARABIDOPSIS THALIANA EXPANSIN-LIKE B1)
At4g28850	XTH26	0.00847	xyloglucan endotransglucosylase/hydrolase 26
At5g04960	AT5G04960	0.00012	putative pectinesterase/pectinesterase inhibitor 46
At5g16910	CSLD2	0.00846	CSLD2 (CELLULOSE-SYNTHASE LIKE D2); cellulose synthase/ transferase, transferring glycosyl groups
At5g57530	XTH12	0.00523	probable xyloglucan endotransglucosylase/hydrolase protein 12
At5g64570	XYL4	0.00021	XYL4; hydrolase, hydrolyzing O-glycosyl compounds / xylan 1,4-beta-xylosidase
At5g64740	CESA6	0.00235	CESA6 (CELLULOSE SYNTHASE 6); cellulose synthase/ transferase, transferring glycosyl groups

Figure S2.

GO enrichment analysis for 465 DEGs identified in the meta-analysis. (A) Full GO enrichment analysis of meta-analysis dataset for biological process. (B) GOslim analysis of enriched categories in the meta-analysis for biological process. (C) Full GO enrichment analysis of meta-analysis dataset for molecular function. (D) GOslim analysis of enriched categories in the meta-analysis for molecular function. Performed with BINGO using hypergeometric test, $p<0.05$ significance threshold and Benjamini and Hochberg FDR correction. The GOfull or GOslim plants ontology file was used. (E) Genes involved in auxin, GA, cytokinin or cell wall metabolism identified in the meta-analysis.

Development • Supplementary information

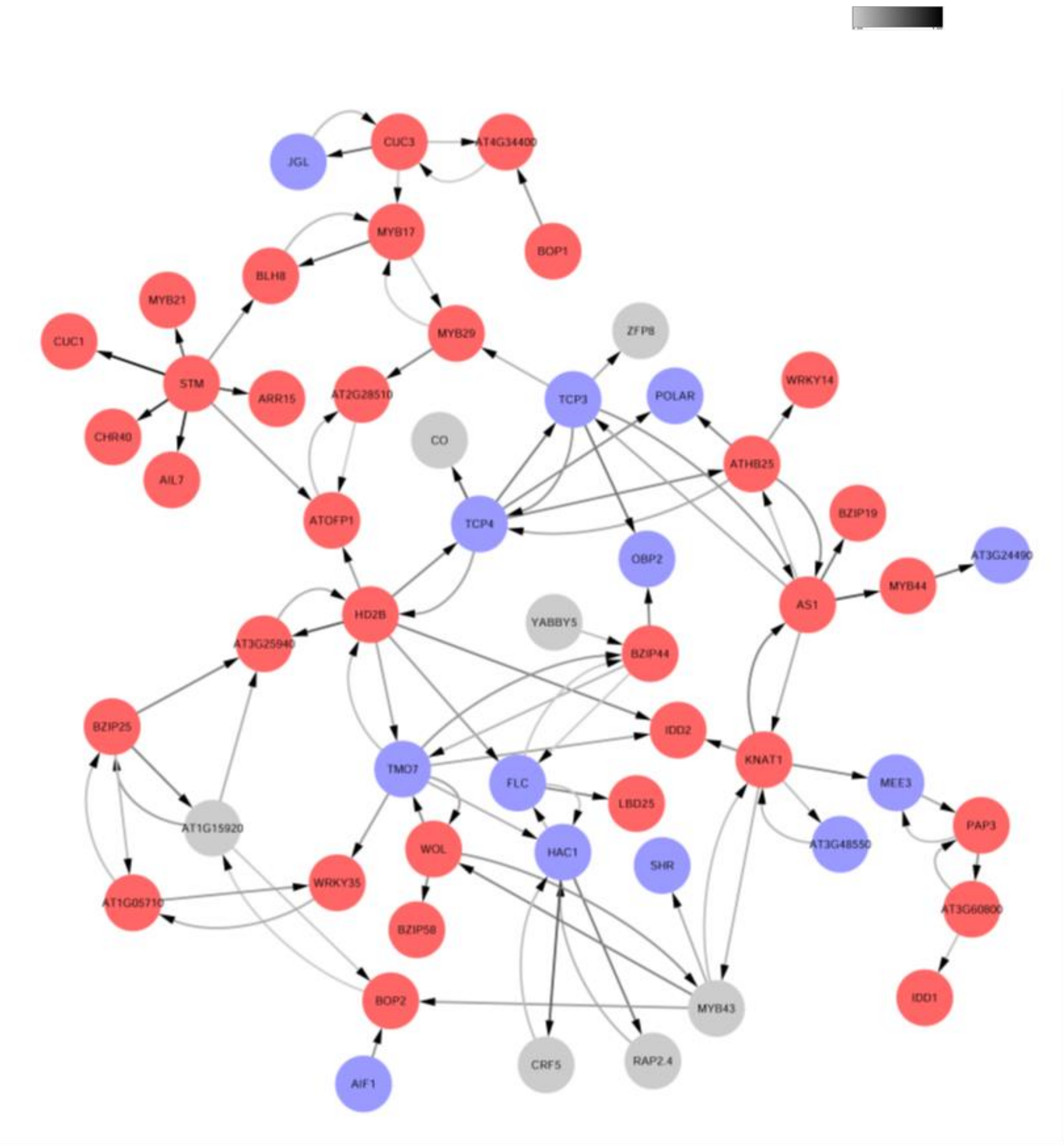

Figure S3.

Directional Bayesian network of 54 transcription-associated DEGs. Nodes in red indicate genes that generally show upregulation in response to increased STM activity, while blue nodes indicate genes showing downregulation. Nodes in grey show differential expression in variable directions across the time-course. Darker edges indicate those predicted to occur more often: 20% (light grey) to 100% (black). The minimum combined edge frequency between any two nodes >=40 % (only edges with a frequency of >20% are shown graphically).

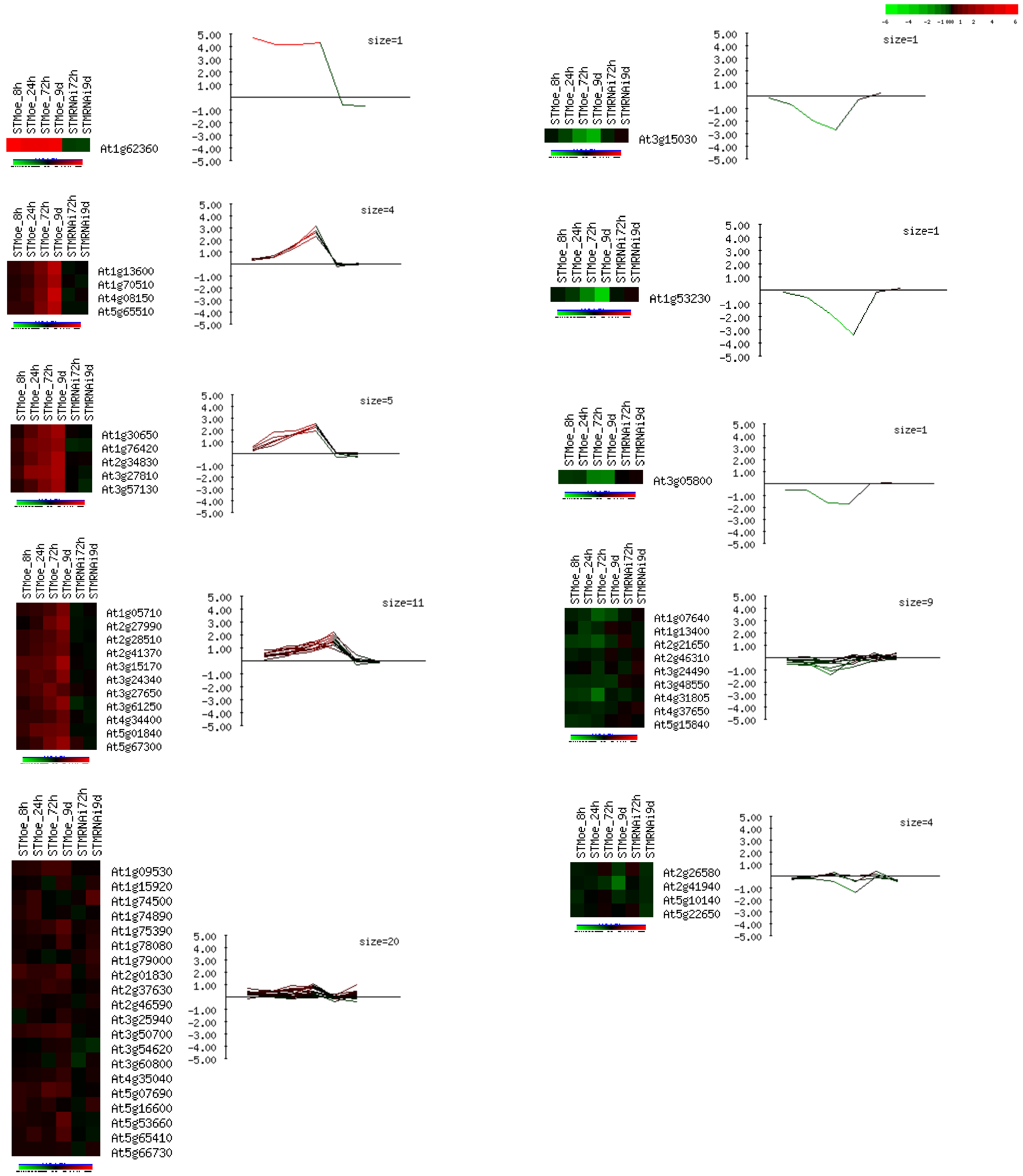

CLUSTER A: At1g62360

CLUSTER B: At1g13600 At1g70510 At4g08150 At5g65510

CLUSTER C: At1g30650 At1g76420 At2g34830 At3g27810 At3g57130

CLUSTER D: At1g05710 At2g27990 At2g28510 At2g41370 At3g15170 At3g24340 At3g27650 At3g61250 At4g34400 At5g01840 At5g67300

CLUSTER E: At1g09530 At1g15920 At1g74500 At1g74890 At1g75390 At1g78080 At1g79000 At2g01830 At2g37630 At2g46590 At3g25940 At3g50700 At3g54620 At3g60800 At4g35040 At5g07690 At5g16600 At5g53660 At5g65410 At5g66730

CLUSTER F: At3g15030

CLUSTER G: At1g53230

CLUSTER H: At3g05800

CLUSTER I: At1g07640 At1g13400 At2g21650 At2g46310 At3g24490 At3g48550 At4g31805 At4g37650 At5g15840

CLUSTER_J: At2g26580 At2g41940 At5g10140 At5g22650

Figure S4.

K-means clustering of 57 transcriptional-regulation-associated DEGs. Clusters reveal DEGs showing similar expression change profiles across the time-course. Red = up-regulation, green = down-regulation. Performed using the EBI EPCLUST tool.

Development • Supplementary information

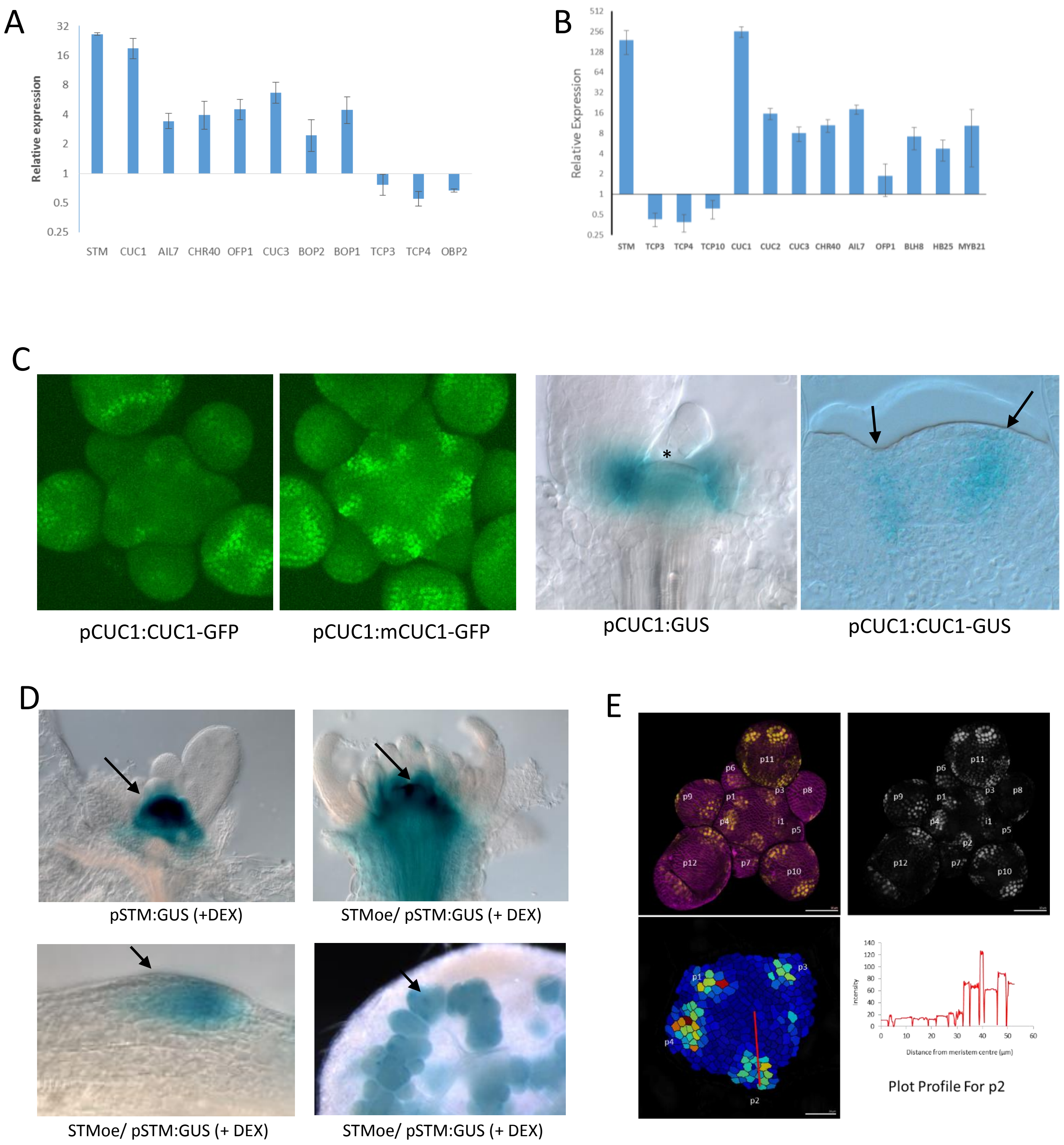

Figure S5. (A) qRT-PCR analysis of expression levels (mRNA abundance) of a subset of network genes 24h after STM induction with DEX. 3 biological replicates were performed. (B) qRT-PCR analysis of expression levels of a subset of network genes in leaves from long-term induced STMoe versus empty vector control line. 3 biological replicates were performed. (C) Expression of CUC1 in the SAM. pCUC1:CUC1-GFP expression is detected weakly in organ boundaries (arrow) while expression of a miR164c-resistant version of CUC1 (pCUC1:mCUC1-GFP) is expressed more broadly (arrow). A CUC1 transcriptional reporter (pCUC1:GUS) is expressed broadly in the shoot apex but the translational fusion (pCUC1:CUC1-GUS) is confined to organ boundaries (arrows). * = approximate centre of SAM dome. (D) Expression of STM in seedlings. A pSTM:GUS reporter is expressed in the SAM (arrow) and shows an expanded domain in STMoe seedlings. pSTM:GUS is also activated during ectopic shoot meristem formation and is sustained thereafter (arrows). (E) Quantification of pDR5::VENUS signal in the inflorescence meristem. Top-left: 2.5D projection of meristem layer 1 produced from confocal stack. VENUS signal = yellow. FM4-64-stained cell membranes = magenta. Developing primordia (p) and incipient primordia (i) are indicated. Top-right: Total VENUS fluorescence in layer one of the SAM. Bottom-left: Segmented image showing cellular compartments extracted from FM4-64 channel. Shading indicates total VENUS signal per cell. Blue = low signal intensity, red = high signal intensity. Red line indicates transect across the meristem-organ boundary. Bottom-right: Quantification of pDR5:VENUS signal intensity across the meristem transect. Troughs indicate the cell boundaries. Scale bars: 20 µm.

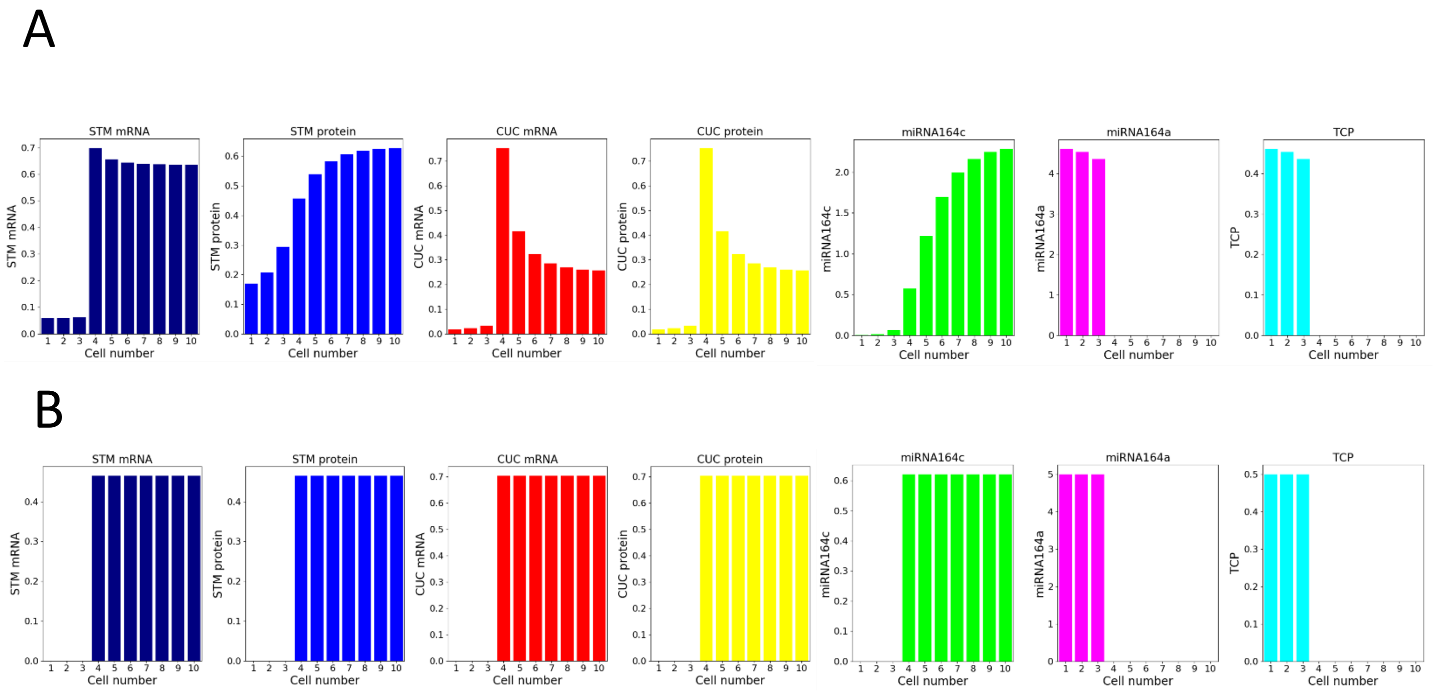

Figure S6.

(A) Baseline ODE model results produced without incorporating STM autoregulation, but with basal STM expression. (B) ODE model results produced with no diffusion of STM.

Development • Supplementary information

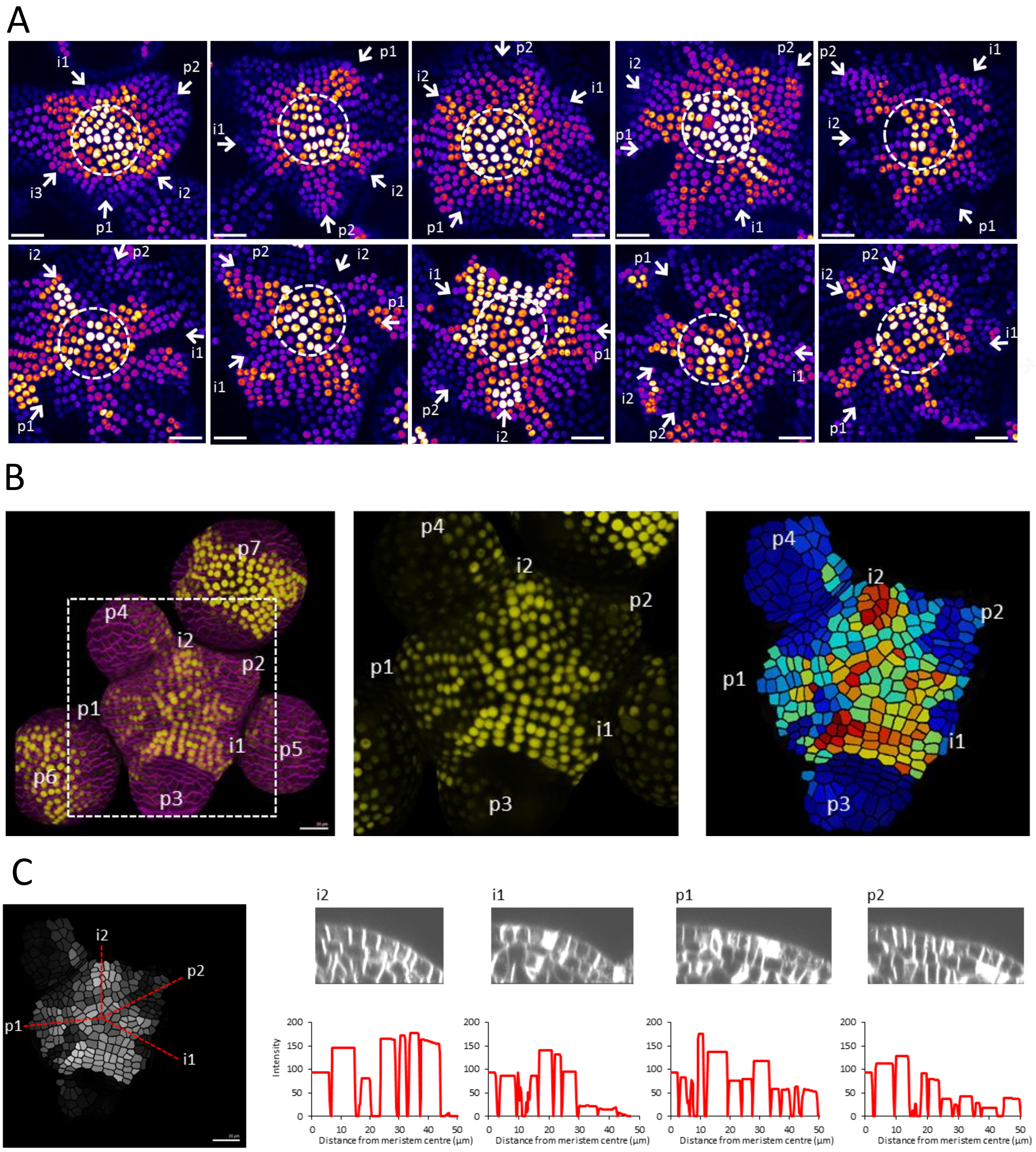

Figure S7. (A) Maximum-intensity projections of pmiR164c:VENUS expression in the SAM obtained by confocal laser scanning microscopy. 10 different apices are shown. Scale bars = 20 µm. (B) Quantification of pmiR164c::VENUS signal in the inflorescence meristem performed as described in Fig. 5. Scale bar = 20 µm (D) Quantification of pmiR164c::VENUS signal intensity across the meristem-organ boundary performed as described in Fig. 5. Scale bar = 20 µm.
